# Tight Regulation of Mechanotransducer Proteins Distinguishes the Response of Adult Multipotent Mesenchymal Cells on PBCE-Derivative Polymer Films with Different Hydrophilicity and Stiffness

**DOI:** 10.3390/cells12131746

**Published:** 2023-06-29

**Authors:** Chiara Argentati, Francesco Morena, Giulia Guidotti, Michelina Soccio, Nadia Lotti, Sabata Martino

**Affiliations:** 1 Department of Chemistry, Biology and Biotechnology, Biochemical and Biotechnological Sciences, University of Perugia, 06122 Perugia, Italy; chiara.argentati@unipg.it (C.A.); francesco.morena@unipg.it (F.M.); 2 Civil, Chemical, Environmental and Materials Engineering Department, University of Bologna, 40131 Bologna, Italy; giulia.guidotti9@unibo.it (G.G.); m.soccio@unibo.it (M.S.); 3 Interdepartmental Center for Industrial Research on Advanced Applications in Mechanical Engineering and Materials Technology, CIRI-MAM, University of Bologna, 40136 Bologna, Italy; 4 CEMIN (Centro di Eccellenza Materiali Innovativi Nanostrutturali per Applicazioni Chimica Fisiche e Biomediche), University of Perugia, 06123 Perugia, Italy

**Keywords:** poly(butylene 1,4-cyclohexane dicarboxylate), copolymerization, mechanical response, cytoskeleton architecture, cell shape, mechanotransduction pathway, hard tissue

## Abstract

Mechanotransduction is a molecular process by which cells translate physical stimuli exerted by the external environment into biochemical pathways to orchestrate the cellular shape and function. Even with the advancements in the field, the molecular events leading to the signal cascade are still unclear. The current biotechnology of tissue engineering offers the opportunity to study in vitro the effect of the physical stimuli exerted by biomaterial on stem cells and the mechanotransduction pathway involved in the process. Here, we cultured multipotent human mesenchymal/stromal cells (hMSCs) isolated from bone marrow (hBM-MSCs) and adipose tissue (hASCs) on films of poly(butylene 1,4-cyclohexane dicarboxylate) (PBCE) and a PBCE-based copolymer containing 50 mol% of butylene diglycolate co-units (BDG50), to intentionally tune the surface hydrophilicity and the stiffness (PBCE = 560 Mpa; BDG50 = 94 MPa). We demonstrated the activated distinctive mechanotransduction pathways, resulting in the acquisition of an elongated shape in hBM-MSCs on the BDG50 film and in maintaining the canonical morphology on the PBCE film. Notably, hASCs acquired a new, elongated morphology on both the PBCE and BDG50 films. We found that these events were mainly due to the differences in the expression of Cofilin1, Vimentin, Filamin A, and Talin, which established highly sensitive machinery by which, rather than hASCs, hBM-MSCs distinguished PBCE from BDG50 films.

## 1. Introduction

Mechanical forces are now acknowledged as a crucial regulator of lifeforms and functions in all aspects of biology [1,2] due to the capability of cells to be sensitive to physical stimuli of different magnitudes at micro- or nanoscale levels and to convert them into biochemical responses [1,2,3]. These molecular pathways, known as mechanosensing and mechanotransduction, are critical for cellular physiology, tissue development, and the maintenance of cell homeostasis and function [4,5,6,7,8]. Both processes involve specific classes of “mechanosensor” proteins that sense external mechanical forces (mechanosensing) and respond by activating intracellular signal pathways (mechanotransduction), resulting in gene expression regulation and, in turn, the control of cellular function(s) [1,2]. At the same time, cells may respond to the above outside-in signaling triggering mechanotransduction pathways in response to specific internal stresses that transmit outside the cells (inside-out signaling) and modify the extracellular matrix, generating a dynamic cross-talk [1,2,4,9]. Thus, understanding the molecular basis of such pathways is critical in elucidating the molecular mechanisms governing cellular functions in physiology and pathology [9,10,11,12,13,14]. Plasma membrane proteins (e.g., ion channels, integrins, cadherins) and intracellular proteins (e.g., focal adhesion complexes, cytoskeleton components, nucleoskeleton components, soluble proteins) are organized in molecular complexes that are spatially juxtaposed to act as players for the mechanosensing and/or mechanotransduction pathways [6,15]. However, how the pathway(s) is (are) activated by one or more physical cues and the protein(s) involved in propagating the signal cascade are still unclear. Therefore, an effort has been made to shed light on how physical stimuli control stem cells’ shapes and functions and to identify the “mechanosensor” proteins involved in these processes.

In this context, designed biomaterials with specific chemical–physical properties are the gold standard in generating suitable ex vivo cell supports, capable of recapitulating the cell microenvironment, and are therefore appropriate to explore the mechanosensing and mechanotransduction pathways [16,17]. Nowadays, biomaterials with designed properties (e.g., surface properties (hydrophylicity/hydrophobicity, micro/nanotopography, unpatterned/patterned microstructures, roughness, ligand availability), mechanical properties (stiffness, elasticity), and electrical properties) have been demonstrated to be capable of modulating the cell shape and function (e.g., migration, proliferation, and differentiation) due to the activation of specific mechanotransduction pathways [14,17,18,19,20,21,22,23,24,25,26,27,28,29,30,31,32,33].

In this work, we have generated a biohybrid system (cells cultured on biomaterials) to study, at a molecular level, the mechanotransduction pathway(s) elicited by biomaterials on multipotent progenitor cells. Understanding these mechanisms is necessary for the development of effective tissue engineering platforms for soft and hard tissues.

We chose mesenchymal/stromal cells (MSCs) as cell sources based on their translational applications with biomaterials and tissue engineering [34,35,36,37,38,39]. MSCs consist of a heterogeneous cell population of stromal cells, comprising multipotent adult stem cells and progenitor cells, that can be isolated from several mesenchymal tissues, such as bone marrow (hBM-MSCs) and adipose tissue (hASCs). Both cells can be easily isolated and cultured in vitro with standardized procedures [34,35,36]. Moreover, they have multipotential properties toward several differentiation lineages and can migrate toward injured sites in response to environmental signals, promoting tissue regeneration through the release of paracrine factors [37,38,40].

We used films of poly(butylene trans-1,4-cyclohexanedicarboxylate) homopolymer (PBCE) to elucidate the mechanotransduction pathway(s) activated by the film properties on hBM-MSCs and hASCs [35,39]. PBCE’s properties can be tuned either by the introduction of increasing amounts of different co-units [41,42,43], such as butylene diglycolate one (BDGx), or by the processing procedure used to generate the polymer films [25].

For instance, the production by a solvent casting procedure of PBCE homopolymer films and PBCE copolymers films containing BDG10 or BDG30 resulted in films with unpatterned microstructures and hydrophilicity that induced a drastic cell shape change, the activation of neuronal markers, and the induction of the neuronal-like differentiation of hBM-MSCs. These effects were absent in hBM-MSCs seeded on homopolymer PBCE films produced with the compression molding procedure [25].

In this work, we used films obtained by the compression molding procedure. We synthesized the P(BCE_50_BDG_50_) (BDG50) copolymer by introducing 50 mol% of BDG co-units into the PBCE homopolymer. The introduction of a flexible BDG co-unit along the PBCE macromolecular chain altered the chemical structure, thereby modifying the hydrophilicity and stiffness of the surface film. The BDG50 film exhibited higher hydrophilicity and reduced stiffness compared to the PBCE film. However, in both films, the stiffness was in the order of MPa; thus, they mimicked the levels of stiffness in hard tissue [44,45,46].

Multipotent mesenchymal/stromal cells were cultured on PBCE and BDG50 films, and both systems were used to study the expression of mechanotransducer proteins involved in the mechanotransduction pathway.

Here, we uncovered a specific mechanotransduction pathway elicited by the stiffness and hydrophilicity properties of PBCE and BDG50 films in multipotent cell types that resulted (i) in the maintenance of the hBM-MSCs’ behavior on the PBCE homopolymer film, and (ii) in the remodeling of the cytoskeleton architecture and cell shape change in hBM-MSCs on the BDG50 copolymer film and in hASCs on both the PBCE and BDG50 films.

## 2. Materials and Methods

### 2.1. Materials and PBCE and BDG50 Polymer Synthesis

Trans-1,4-cyclohexanedicarboxylic acid (CHDA; Fluorochem, Hadfield, Derbyshire, UK), diglycolic acid (DGA; Merck KGaA, Darmstadt, Germany), 1,4-butanediol (BD; Merck KGaA, Darmstadt, Germany), and titanium IV butoxide (TBT; Merck KGaA, Darmstadt, Germany) were all used as purchased.

**Figure 1:**
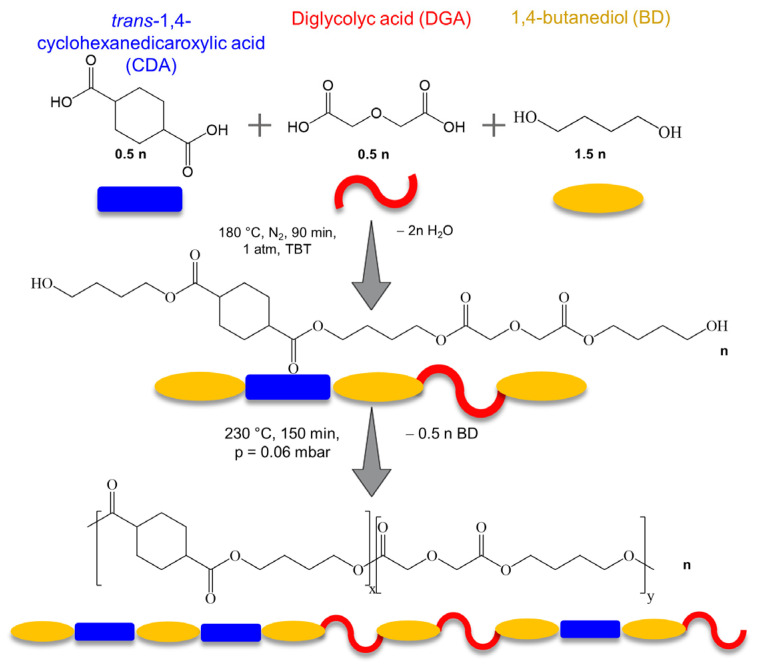
Schematic representation of the synthetic procedure of PBCE polymer and BDG50 copolymer.

Poly(butylene *trans*-1,4-cyclohexanedicarboxylate) (PBCE) and poly(butylene *trans*-1,4-cyclohexane dicarboxylate/diglycolate) random copolymer (BDG50), containing 50 mol% of butylene diglycolate co-units, were synthesized by two-step polycondensation in the melt, according to our previous studies [25]. A schematic representation of the synthetic procedure is reported in the scheme in Figure 1. The presence of an equimolar amount of BDG moieties, containing ether oxygen atoms, allowed us to enhance both the surface hydrophilicity and the flexibility of the final material, maintaining at the same time a suitable amount of the crystalline phase, the key requirement for the processing of the sample in the form of a film, as described below. After the synthesis, both polymers were purified by dissolution in chloroform and further precipitation in methanol.

### 2.2. Production of PBCE and BDG50 Polymer Films

Free-standing films of PBCE and BDG50 were prepared by compression molding (Carver C12 laboratory press). The purified polymers were placed between two Teflon sheets and heated at a temperature 40 °C higher than the melting point of the materials. After complete melting, a pressure of 5 ton/m^2^ was applied for 2 min. Then, the films were ballistically cooled to room temperature in the press.

### 2.3. PBCE and BDG50 Polymer Film Characterization

Both films were characterized from chemical, thermal, wettability, and mechanical points of view. In detail, proton nuclear magnetic resonance (^1^H-NMR, Varian Inova 400-MHz, Varian Associates, Palo Alto, CA, USA) was used to confirm the chemical structure and, in the case of the copolymer, the effective chemical composition. The molecular weight (M_n_) and polydispersity index (Ð) were evaluated by means of gel permeation chromatography (GPC, HPLC 1100 apparatus equipped with a PLgel 5-mm MiniMIX-C column and an RI detector). The surface wettability was investigated by static water contact angle (WCA) measurements (optical contact angle and surface tension meter CaAM 101, KSV Instruments Ltd., Helsinki, Finland) carried out on flat film surfaces. Regarding thermal characterization, the thermal stability was measured by thermogravimetric analysis (TGA, PerkinElmer TGA4000, Shelton, CT, USA), carried out under a nitrogen flow, at a heating rate of 10 °C/min, in the temperature range 40–800 °C. The main thermal transitions were determined by differential scanning calorimetry (DSC, Perkin Elmer DSC6, Shelton, CT, USA) under a nitrogen flow, using the following conditions: heating at 20 °C/min from −60 °C to 180 °C (I scan), holding for 3 min, quenching to −60 °C (100 °C/min), and heating again to 180 °C at 20 °C/min (II scan). Mechanical characterization was performed through stress–strain measurements (Instron 5966), carried out on polymeric film stripes: each specimen (gauge length of 2 cm) was stretched at a constant rate of 10 mm/min until break, and the values of Young’s modulus (E), stress at break (σ_B_), and elongation at break (ε_B_) were calculated from the obtained stress–strain curves.

### 2.4. Human Adult Mesenchymal/Stromal Multipotent Cells’ Isolation and In Vitro Culture

Adult mesenchymal/stromal multipotent cells were available in our laboratory for research activity only [30,47,48]. Both cell types were isolated from waste samples; thus, hBM-MSCs were collected from bone marrow thanks to the washout of the femur medullary cavities of adult donor subjects undergoing primary total hip replacement. hASCs were collected from biopsy adipose tissue during aesthetic intervention. In both cases, the technique of isolating human adult mesenchymal/stromal multipotent cells was sporadic and not part of a specific project, and all treatments were carried out with the agreement of donors (60 years old) and in compliance with the Helsinki Declaration.

hBM-MSCs were isolated from the mononuclear cells from bone marrow using Lympholyte (Cedarlane Laboratories Limited, Hornby, ON, Canada), containing the heterogeneous mesenchymal stromal population, and were seeded in culture flasks in growth medium consisting of Dulbecco’s Modified Eagle Medium (DMEM) (Euroclone S.p.A., Pero (MI), Italy), containing heat-inactivated fetal bovine serum (FBS) 10%, 2 mM L-glutamine, and 1% penicillin–streptomycin (Euroclone S.p.A., Pero (MI), Italy) in a humidified atmosphere and at 5% CO_2_. The non-adherent cells were removed after 5 to 7 days, and fresh medium was introduced to the flasks every three days. A fibroblast-like colony began to form after 15 days.

For hASC isolation, adipose tissue was incubated for 40 min at 37 °C and 5% CO_2_, with 0.075% collagenase from Clostridium (Sigma Aldrich, St. Louis, MO, USA) for tissue digestion, prepared in PBS containing 0.5% bovine serum albumin (BSA). The digestate was centrifuged twice and the cell pellet was then re-suspended in a growth medium, RPMI-1640 (Euroclone S.p.A., Pero (MI), Italy), supplemented with 10% FBS, 1% L-glutamine, and 1% penicillin–streptomycin, plated in tissue culture flasks (TCP), and incubated at 37 °C, 5% CO_2_.

Human adult mesenchymal/stromal multipotent cells expressing human anti-CD44, -CD73, -CD90, and -CD105 were used, as well as anti-CD45, -CD34, human leukocyte antigen (HLA)-ABC, CD (BD Biosciences). The FlowJo software (Tree Star, Ashland, OR, USA) was employed to analyze the data, and cells were electrically gated based on their light-scattering characteristics to distinguish between cell debris and cells. The negative control was composed of isotype-matched non-specific antibodies [30,47,48]. Moreover, both hBM-MSCs and ASCs were able to differentiate toward osteogenic, adipogenic, and neural lineage [17,23,24,25,30,39,47,48].

### 2.5. Culture of hBM-MSCs and hASCs on Polymer Films

Each film was cut into a 1 cm^2^ square before being sterilized for 30 s in 70% ethanol, washed with sterile PBS, and then deposited on multi-well plates. After drying, a suspension of human adult mesenchymal/stromal multipotent cells was sown drop by drop onto sterile films. The growth culture medium was gradually added to each film after 45 min. Cells plated on films were incubated at 37 °C and 5% CO_2_ in a humidified environment following the canonical culture conditions. The medium was replaced every three days, and cell cultures were examined for cell viability, morphology, and mechanotransduction molecular pathway activation.

### 2.6. Adhesion of hBM-MSCs and hASCS on PBCE and BDG50 Films

First, 3 × 10^3^ cells were seeded on PBCE and BDG50 films and on CTR (glass coverslip (GC)). The time-course adhesion of both cell types on the polymer films and CTR was evaluated at the time of seeding (t = 0 min), as well as after t = 45 min and t = 2.5 h of cell seeding. Images were captured with a Canon digital camera (PowerShot G10, Canon, Tokyo, Japan) and a brightfield microscope (Eclipse-TE2000-S, Nikon, Tokyo, Japan). The cell adhesion on PBCE and BDG50 was tested also at day 3 by staining with Phalloidin and Vinculin. Fluorescence images were captured with a fluorescence microscope (Eclipse-TE2000-S, Nikon, Tokyo, Japan) equipped with an F-ViewII FireWire camera (Soft Imaging System, Olympus, Münster, Germany).

### 2.7. Viability Assay

At different time intervals (3, 7, 14, and 21 days), 3 × 10^3^ cells seeded on PBCE and BDG50 (1 cm^2^ square) were evaluated to assess their vitality. In tests, the same number of cells was seeded on tissue culture polystyrene (TCP) as an internal control. According to the recommendations provided by the manufacturer, 3-(4,5-dimethylthiazol-2-yl)-2,5-diphenyltetrazolium bromide (MTT) (Sigma-Aldrich, St. Louis, MO, USA) was added to cell–film cultures to test the viability of cells. Experiments were carried out using cells seeded on TCP as an internal control. Interference effects of polymer films without cells on the MTT assay were also considered. A microtiter plate reader (ELISA reader, DV990BV6, GDV, Roma, Italy) was used to measure the absorbance of the samples at 589 nm, with a reference wavelength of 650 nm.

### 2.8. Cell Proliferation

Cell proliferation was evaluated by seeding 3 × 10^3^ cells on PBCE and on BGD50 films (1 cm^2^ square) at different time points (3, 7, 14, and 21 days). As an internal control, experiments were performed by seeding the same number of cells on TCP.

The proliferation assay was performed using the Invitrogen^TM^ Countess™ Automated Cell Counter (Thermo Fisher, Invitrogen™, Grand Island, NY, USA) and the procedure was performed according to the manufacturer’s recommendation for adherent cells. The use of Trypan Blue Solution, 0.4% (Invitrogen™, Grand Island, NY, USA), was recommended in the method.

### 2.9. Immunofluorescence

Immunofluorescence was carried out as previously described [17,25,28,49]. In brief, cells seeded on PBCE and BDG50 squares, as well as on the glass coverslip (GC), as a negative control, were washed twice with PBS, fixed for 20 min in 4% paraformaldehyde, washed twice with PBS, and then permeabilized (PBS + 3% FBS, 0.5% Triton X-100) and blocked (PBS + 3% FBS, 0.05% Triton X-100) for 1 h at room temperature. Samples were either treated overnight at 4 °C with the primary human antibody anti-β-Tubulin (1:200, Elabscience, Houston, Texas, USA) for the staining of microtubules, anti-Vimentin (1:200, Cell Signaling Technology, Danvers, MA, USA) for the staining of intermediate filaments, and anti-Vinculin (1:150, Abcam, Cambridge, UK) for the staining of focal adhesion spots, or for 20 min at room temperature with Phalloidin (Alexa Fluor 488 Phalloidin, Invitrogen, Grand Island, NY, USA) for F-Actin staining. In case of microtubules, Vimentin, and Vinculin, after washes with PBS, samples were incubated with secondary antibodies, namely donkey anti-mouse Alexa Fluor 594-nm conjugated (Invitrogen, Grand Island, NY, USA) or donkey anti-rabbit Alexa Fluor 594-nm conjugated (Invitrogen, Grand Island, NY, USA), for 1 h at 37 °C. Samples were mounted and nuclei were counterstained with Vectashield with DAPI (Vector Laboratories, Inc., Burlingame, CA, USA) after being washed with PBS. Images were acquired using fluorescence microscopy (Eclipse-TE2000-S, Nikon, Tokyo, Japan) equipped with an F-ViewII FireWire camera (Soft Imaging System, Olympus, Münster, Germany). Interference from a fluorescence microscope for PBCE and BDG50 without cells was evaluated.

### 2.10. Computational Imaging Analysis

Cyto-morphometric descriptors, i.e., the cellular shape index (CSI), eccentricity, roundness, solidity, spread area, and spread index, were measured on 100 digital images of hASCs and hBM-MSCs on CTR, PBCE, and BDG50 stained with Phalloidin-GREEN and β-Tubulin-RED. The nuclear area, nuclear shape index, and nuclear roundness were measured on 100 digital images of hASCs and hBM-MSCs on CTR, PBCE, and BDG50 stained with DAPI (Vector Laboratories, Inc., Burlingame, CA, USA). The analysis was performed using Fiji (Fiji Life-Line, v. 2015, U.S. National Institutes of Health, Bethesda, Maryland, USA) as previously reported [2,50,51].

### 2.11. Cell Extracts

Cells were detached from films and TPC after 7 days of culture by incubation with trypsin. After being cleaned in PBS, cell pellets were re-suspended in 10 mM sodium phosphate buffer, pH 6.0, adding 0.1% (*v*/*v*) Nonidet NP40 detergent (Sigma-Aldrich, St. Louis, MO, USA) [52]. Three rounds of sonication were performed on the cell lysates. The procedures were performed at 4 °C. Protein concentrations were determined using the Bradford assay with BSA as the standard [28,49,53].

### 2.12. Western Blotting

Protein extracts (35µg for each sample) of TCP, PBCE, and BDG50 were separated by SDS-PAGE and Western blotting, and then immunodetected with the primary antibodies (listed in Table 1) and one of the following secondary antibodies: anti-mouse IgG, HRP-linked antibody (Cell Signaling Technology, Danvers, MA, USA) anti-rabbit IgG, HRP-linked antibody (Cell Signaling Technology, Danvers, MA, USA), and rabbit anti-goat IgG antibody, HRP-conjugated (Sigma Aldrich, St. Louis, MI, USA). The immunostaining procedures were carried out using the ECL^TM^ Detection System (GE Healthcare, Fairfield, CT, USA).

Densitometry analyses were conducted using the Fiji software (Fiji Life-Line, v. 2015, National Institutes of Health, Bethesda, MD, USA). Relative band intensities were normalized to Actin as an internal reference. Results are expressed as the mean ± SD of three independent experiments.

### 2.13. Venn Diagram Analysis

VennPlex version 1.0.0.2 [54] was utilized in the creation of Venn diagrams. VennPlex offers the capability to determine specific factors that are upregulated, downregulated, or contra-regulated (regulated in the opposite direction) in relation to their corresponding control groups. The datasets utilized to generate the diagrams were obtained by converting protein expression levels into log2-fold change values relative to the control cell system.

### 2.14. Statistical Analysis

Data analyses were reported as the mean ± SD and median with interquartile range (GraphPad 8.0 Software, San Diego, CA, USA). Parametric (one-way ANOVA) or non-parametric tests (Kruskal–Wallis) followed by post-tests were used according to datasets. *p* ≤ 0.05 was considered statistically significant. The statistical test used is indicated in the legend of each figure.

## 3. Results

**Figure 2:**
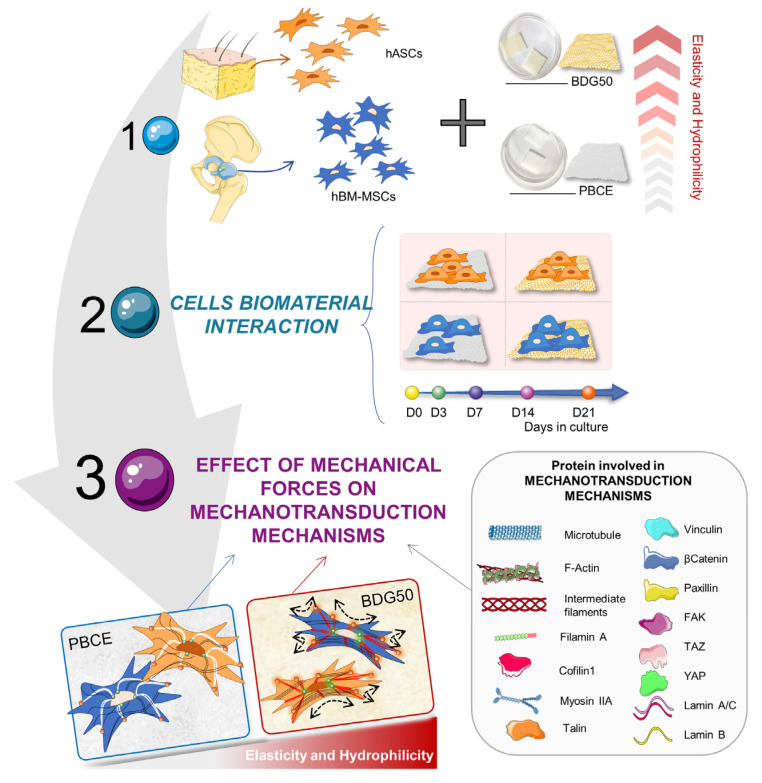
Experimental plan. Step 1 illustrates the types of human adult mesenchymal/stromal multipotent cells used for the study, and the films: PBCE and BDG50 polymer-produced films. Step 2 shows the interactions of hBM-MSCs and hASCs with PBCE and BDG50 films and their culture for 21 days. In Step 3, the mechanotransducer proteins investigated are listed.

In this work, we studied the mechanotransduction pathways activated by PBCE homopolymer and BDG50 copolymer films on hBM-MSCs and hASCs. The workflow, illustrated in Figure 2, started with the polymers’ ad hoc design and synthesis, followed by the production and characterization of the PBCE and BDG50 films, and then the in vitro culture of both hBM-MSCs and hASCs on the polymer films (Step 1). Then, it continued with the evaluation of the suitability of PBCE and BDG50 films for the long-term culture of hBM-MSCs and hASCs (Step 2) and the investigation of the effects of PBCE and BDG50 on the mechanotransduction pathways in hBM-MSCs and hASCs (Step 3). Immunofluorescence, computational imaging analysis, and Western blotting were performed to study the protein expression involved in the mechanotransduction pathway.

### 3.1. PBCE and BDG50 Film Characterization

**Figure 3:**
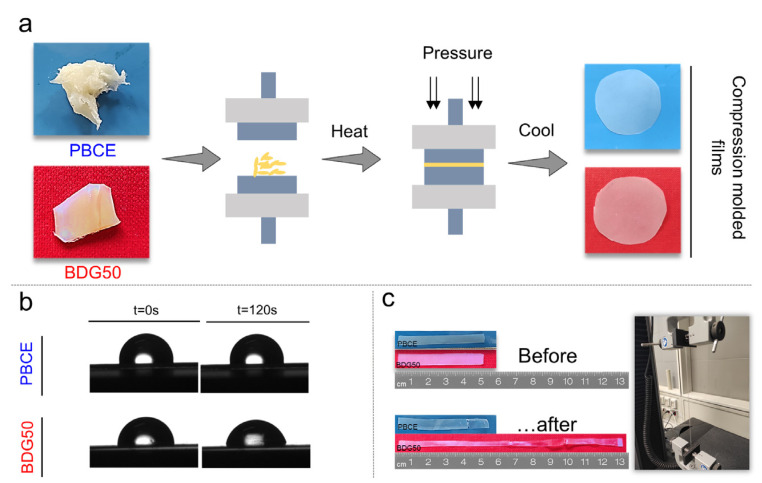
(**a**) Schematic representation of the realization of films by compression molding; (**b**) different WCA and (**c**) different mechanical behavior of PBCE and BDG50 films.

According to our previous study, the chemical structures of PBCE and BDG50 were confirmed by ^1^H-NMR [25]. The chemical composition of the BDG50 sample was also calculated, resulting in a very close-to-feed one. The molecular weights were high and comparable (50,300 and 47,500 Da, respectively) with polydispersity indexes of 1.7 and 1.5, respectively. All these results indicate good control over the polycondensation process. The materials were then compression-molded into films (Figure 3a) with a thickness of approximately 150 µm and characterized from the solid-state point of view.

The prepared films differed in their hydrophilicity. Copolymerization was responsible for an increase in surface wettability (lower hydrophobicity), due to the presence of ether oxygen atoms. Thus, the water contact angle (WCA) values immediately after drop deposition were 100° for PBCE and 91° for BDG50, and after 120 s, the evolution of the drop shape in BDG50 gave a WCA value of approximately 77°, whereas the PBCE WCA was unchanged (Figure 3b).

Copolymerization did not remarkably affect the thermal stability. In fact, the onset temperature (T_onset_) was above 350 °C in both cases, although slightly lower for the copolymer (366 vs. 395 °C for PBCE) due to the presence of ether oxygen atoms, which are more prone to thermo-oxidative processes, in agreement with previous results [25].

Differential scanning calorimetry (DSC) revealed that both films were semicrystalline (the calorimetric traces of both polymer films showed a melting endothermic peak) with a glass transition step associated with the sample’s amorphous portion below room temperature. Both the copolymer melting enthalpy (∆H_m_) and melting temperature T_m_ were lower with respect to PBCE: T_m_ decreased from 165 °C to 84 °C, while the melting enthalpy shifted from 29 J/g to 20 J/g. Such results are a consequence of the decreased crystallization capability of BCE moieties (lower ∆H_m_) in the copolymer, together with the formation of crystals with a lower degree of perfection (lower T_m_), due to PBCE’s chemical modification through copolymerization. Lastly, the glass transition temperature (T_g_), measured after rapid cooling from the molten state, decreased from 15 °C for PBCE to −20 °C for BDG50, indicating that, in both samples, the amorphous phase was rubbery but was more mobile in the copolymer [25].

Mechanical characterization, performed via stress–strain measurements, revealed different behavior for the two materials (Figure 3c). Once again, the insertion of ether oxygen atoms along the PBCE main chain allowed us to modify significantly, and in a controlled way, the homopolymer’s mechanical performance. In detail, the Young’s modulus decreased by more than five times (E_PBCE_ = 560 MPa; E_BDG50_ = 94 MPa) with copolymerization, whereas the elongation at break increased from only 40% for PBCE to almost 500% for BDG50. Lastly, the stress at break decreased due to copolymerization, shifting from a value of 27 MPa for the homopolymer to 6 MPa for the copolymer [25].

The overall data indicated that the BDG50 copolymer film was more hydrophilic and flexible (reduced stiffness) than the PBCE homopolymer film.

### 3.2. PBCE and BDG50 Are Suitable for the Long-Term Culture of hBM-MSCs and hASCs

**Figure 4:**
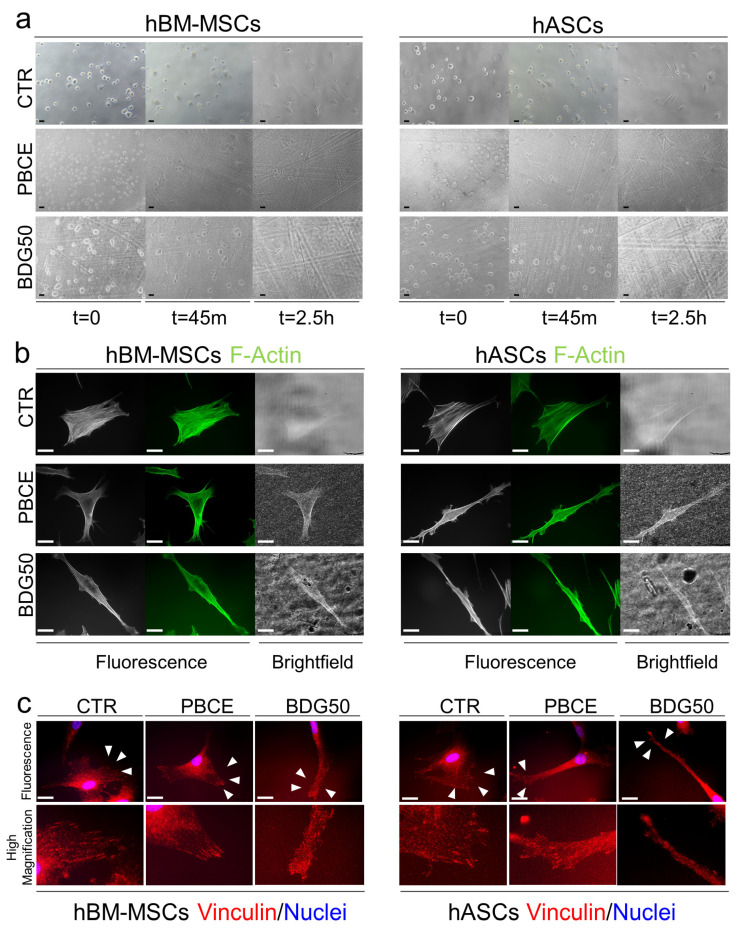
hBM-MSC and hASC adhesion on CTR, PBCE, and BDG50 films. (**a**) Representative brightfield images of hBM-MSC and hASC adhesion at t = 0 (after seeding), after 45 min (t = 45 min), and after 2.5 h (t = 2.5 h) on PBCE and BDG50 films and CTR system, obtained with the microscope Eclipse-TE2000-S (Nikon) equipped with a Nikon digital sight DS-L1 (NIKON, Tokyo, Japan). Scale bar = 100 µm. (**b**) Representative fluorescence images of F-Actin (GREEN) in hBM-MSCs and hASCs on PBCE and BDG50 films and CTR system at D3, and relative brightfield images, obtained with the fluorescence microscope Eclipse-TE2000-S (Nikon, Tokyo, Japan) equipped with an F-ViewII FireWire camera (Soft Imaging System, Olympus, Münster, Germany). Scale bar = 20 µm. (**c**) Representative fluorescence images of Vinculin of hBM-MSCs and hASCs on PBCE and BDG50 films and CTR system at D3, and relatively high-magnification digital images, obtained with a fluorescence microscope (Eclipse-TE2000-S, Nikon, Tokyo, Japan) equipped with an F-ViewII FireWire camera (Soft Imaging System, Olympus, Münster, Germany). Scale bar = 20 µm.

First, we assessed the adhesion of hBM-MSCs and hASCS on the PBCE and BDG50 films. Representative images revealed the time-course adhesion of both cell types on the polymer films and control system (CTR) at the time of seeding (t = 0 min), and after t = 45 min and t = 2.5 h of cell seeding, when the majority of both cell types had adhered to the film surfaces and to the CTR (Figure 4a). The cell adhesion on PBCE and BDG50 was comparable to the related counterpart on the CTR, as revealed in the culture at day 3 by the representative brightfield images, Phalloidin staining, and the high magnification of the Vinculin focal adhesion spot immunostaining [55] (Figure 4b,c).

**Figure 5:**
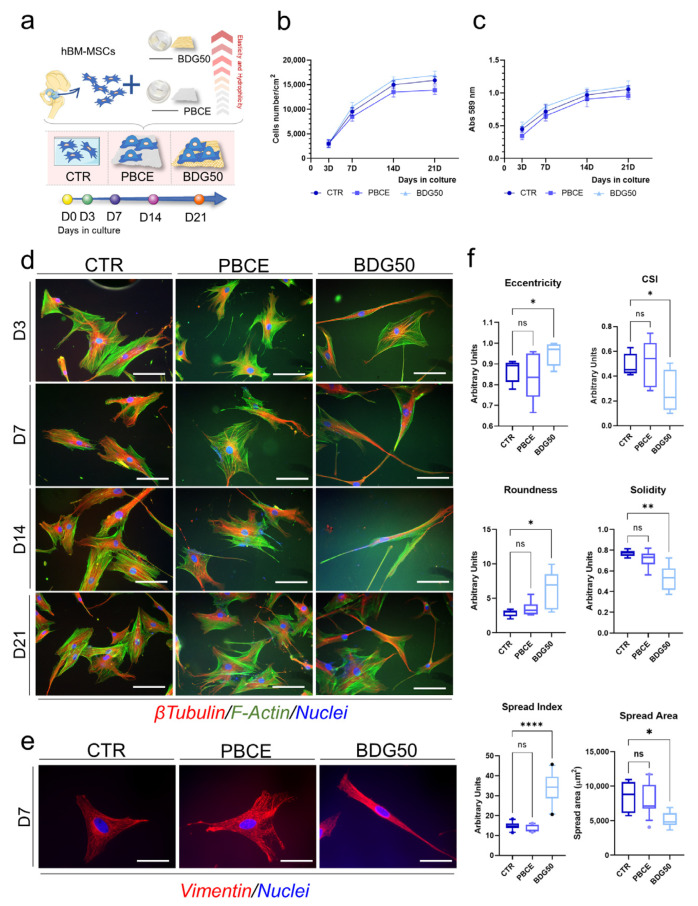
Culture of hBM-MSCs on PBCE and BDG50 films. (**a**) Schematic representation of the long-term culture of hBM-MSCs on PBCE and BDG50 films, and in the control conditions (CTR). (**b**) Cell proliferation and (**c**) cell viability on PBCE, BDDG50 films, and CTR at different time points (D3, D7, D14, and D21). (**d**) hBM-MSCs’ morphology during the time in culture. Representative images of F-Actin (GREEN), microtubules (RED), and nuclei (BLUE) on PBCE, BD50, and CTR. Scale bar = 100 µm. (**e**) Representative images of Vimentin (RED) and nuclei (BLUE) on PBCE, BD50, and CTR. Scale bar = 20µm. (**f**) Computational imaging analysis of hBM-MSCs on PBCE, BDG50, and CTR: eccentricity, cell shape index (CSI), roundness, solidity, spread area, and spread index. Results are expressed as mean ± SD of three independent experiments in (**b**,**c**); represented as box and whiskers (min to max) in (**e**) with Kruskal–Wallis test and Dunn’s multiple comparison test. * *p* < 0.05, ** *p* < 0.01, **** *p* < 0.0001, ns: not significant.

**Figure 6:**
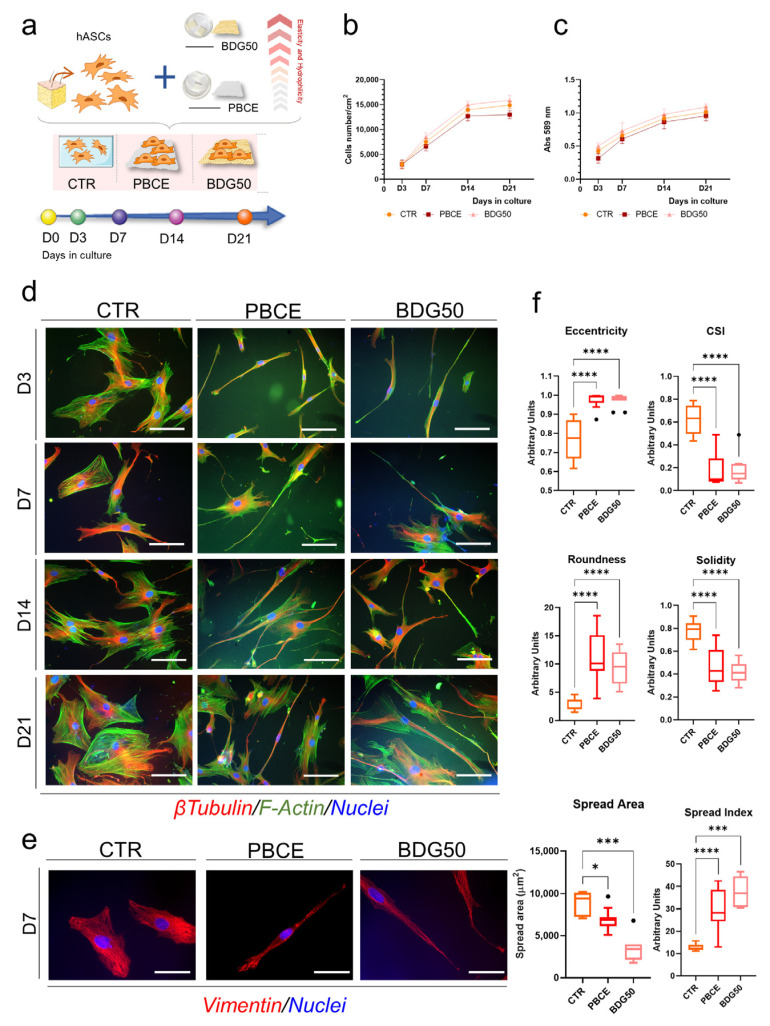
Culture of hASCs on PBCE and BDG50 films. (**a**) Schematic representation of the long-term culture of hBM-MSCs on PBCE and BDG50 films, and in the control conditions (CTR). (**b**) Cell proliferation and (**c**) cell viability on PBCE, BDDG50, and CTR at different time points (D3, D7, D14, and D21). (**d**) hASCs’ morphology during the time in culture. Representative images of F-Actin (GREEN), microtubules (RED), and nuclei (BLUE) on PBCE, BD50, and CTR. Scale bar = 100 µm. (**e**) Representative images of Vimentin (RED) and nuclei (BLUE) on PBCE, BD50, and CTR. Scale bar = 20 µm. (**f**) Computational imaging analysis of hASCs on PBCE, BDG50, and CTR: eccentricity, cell shape index (CSI), roundness, solidity, spread area, and spread index. Results are expressed as mean ± SD of three independent experiments in (**b**,**c**); represented as box and whiskers (min to max) in (**e**) with Kruskal–Wallis test and Dunn’s multiple comparison test. * *p* < 0.05, *** *p* < 0.001, **** *p* < 0.0001.

Next, we determined whether the PBCE and BDG50 films were suitable for the culture of both hBM-MSCs and hASCs. We performed long-term culture investigations (21 days (D)) by seeding multipotent cells on polymer films in the growth culture medium and assessed the cell proliferation, viability, and morphology (Figure 5 and Figure 6). As the experimental control (CTR), human adult mesenchymal/stromal multipotent cells were seeded on tissue culture polystyrene or on glass coverslips in the growth culture medium (Figure 5a and Figure 6a). Cultures were monitored at different time points (D3, D7, D14, D21).

We found a comparable cell proliferation rate for hBM-MSCs and hASCs cultured on PBCE and BDG50 films and on the CTR system (Figure 5b and Figure 6b). We also found a similar dehydrogenase activity curve in both cell types on the PBCE and BDG50 polymer films with respect to the control system, indicating the absence of cell cytotoxicity (Figure 5c and Figure 6c). Moreover, all cell–polymer cultures were free of pyknotic signs (Figure 5d and Figure 6d, representative images). Both hBM-MSCs and hASCs grew adherent to the PBCE and BDG50 films and to the CTR (Figure 4, Figure 5d and Figure 6d).

These results demonstrate the biocompatibility of PBCE and BDG50 for both hBM-MSC and hASC cultures.

The cell morphology was analyzed by evaluating the architecture of the cytoskeleton via the immunostaining of F-Actin fibers, microtubules, and the intermediate filaments of Vimentin (Figure 5d,e and Figure 6d,e) [56,57].

hBM-MSCs on PBCE maintained the canonical mesenchymal fibroblast-like morphology, as in the control system (Figure 5d,e). However, when cultured on the BDG50 film, hBM-MSCs showed a thin, elongated shape, as revealed by F-Actin, microtubule (Figure 5d,e, representative images), and Vimentin organization (Figure 5e, representative images at D7).

The cells’ morphology was observed to be different after day 3 of the culture and was maintained throughout the culture period (D21; Figure 5d).

The computational cyto-morphometric analysis validated these results (Figure 5f). We found that the descriptors of eccentricity, cell scape index (CSI), roundness, solidity, spread area, and spread index (calculated as the mean value for time points 3, 7, 14, and 21 days) followed a similar trend in hBM-MSCs on PBCE and CTR compared to cells on BDG50, where all indicators were different (Figure 5f). Herein, the eccentricity, roundness, and spread index were consistently increased, whereas the CSI, solidity, and spread area were decreased, compared to the PBCE and CTR cultures, where the levels of the indicators were comparable (Figure 5f).

In comparison to hBM-MSCs, hASCs demonstrated changes in cell shape on both PBCE and BDG50 films (Figure 6d,e, representative images). The cytoskeleton architecture of hASCs on both polymer films reorganized and was significantly elongated, compared to the mesenchymal fibroblast-like shape of the control cells (Figure 6d,e). The new cell shape was evident at D3 and was maintained over time in the culture (Figure 6e).

Again, these results were confirmed by the computational cyto-morphometric analysis, which, however, revealed an interesting mirroring trend (Figure 6f). All indicators (eccentricity, CSI, roundness, solidity, cell spread area, and spread index) were comparable among hASCs cultured on PBCE and BDG50, whereas both were different toward cells cultured on the CTR (Figure 6f). In detail, compared to the CTR cultures, in the PBCE and BDG50 cultures, the eccentricity, roundness, and spread index increased, whereas the CSI, solidity, and spread area decreased (Figure 6f).

#### 3.2.1. hBM-MSCs and hASCs Respond Differently to the Chemical and Physical Properties of PBCE and BDG50

**Figure 7:**
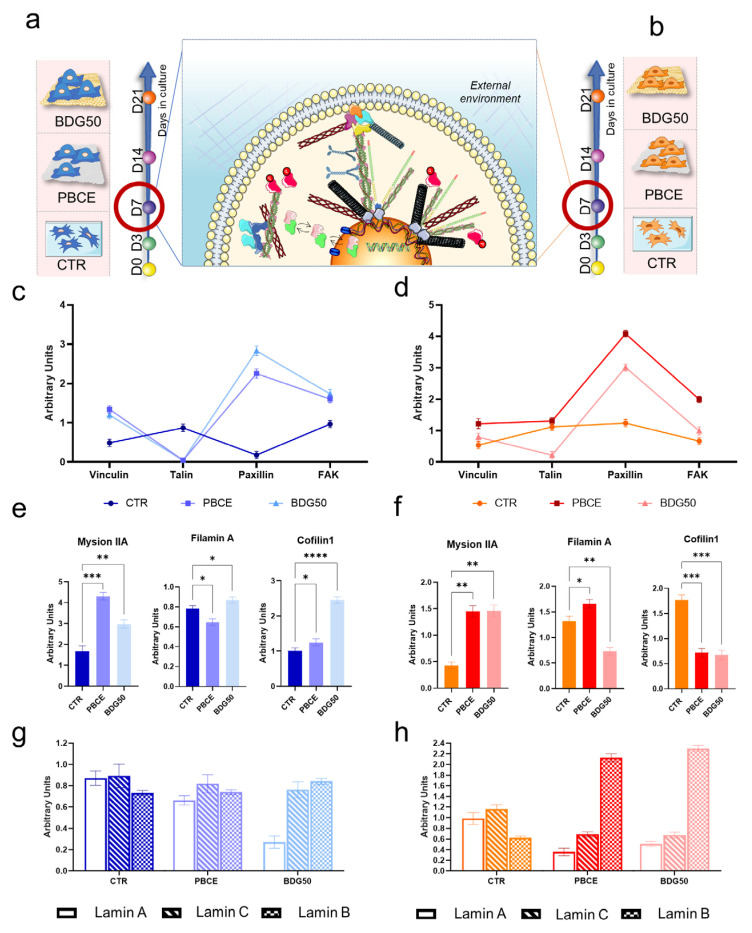
Expression of proteins involved in mechanotransduction mechanism in hBM-MSCs (**a**,**c**,**e**,**g**) and hASCs (**b**,**d**,**f**,**h**) after 7 days of culture on films of PBCE and BDG50 and CTR. (**a**,**b**) Schematic representation of the experimental plan of hBM-MSCs and hASCs on TCP (CTR), PBCE, and BDG50 films. (**c**,**d**) FA protein expression profile in hBM-MSCs (**c**) and hASCs (**d**). Western blotting bands, densitometric analysis, and relative statistical analysis are reported in Supplementary Figures S1 and S3. (**e**,**f**) Densitometric analysis of Actin-linking proteins Myosin IIA, Filamin A, and Cofilin1 in hBM-MSCs (**e**) and hASCs (**f**). Western blotting bands are reported in Supplementary Figure S4a. (**g**,**h**) Expression of Lamin A, Lamin C, and Lamin B in hBM-MSCs (**g**) and hASCs (**h**). Western blotting bands, densitometric analysis, and relative statistical analysis are reported in Supplementary Figure S6. All results are expressed as mean ± SD of three independent experiments. * *p* < 0.05, ** *p* < 0.01, *** *p* < 0.001, **** *p* < 0.0001.

**Figure 8:**
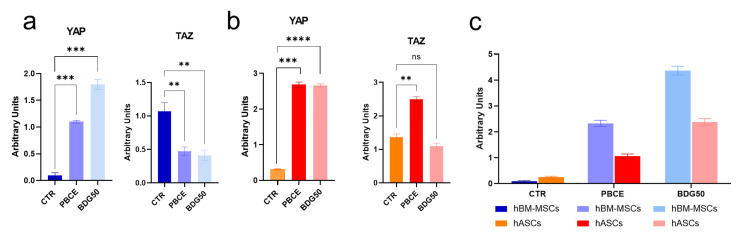
Mechanotransducer transcription factors. (**a**,**b**) Densitometric analysis of mechanotransducer transcription factors YAP and TAZ in hBM-MSCs (**a**) and in hASCs (**b**) on PBCE, BDG50, and CTR systems. (**c**) YAP/TAZ ratio in hBM-MSCs and hASCs on PBCE, BDG50, and CTR systems. Relative bands are reported in Supplementary Figure S7. Results are expressed as mean ± SD of three independent experiments. ** *p* < 0.01, *** *p* < 0.001, **** *p* < 0.0001, ns: not significant.

**Figure 9:**
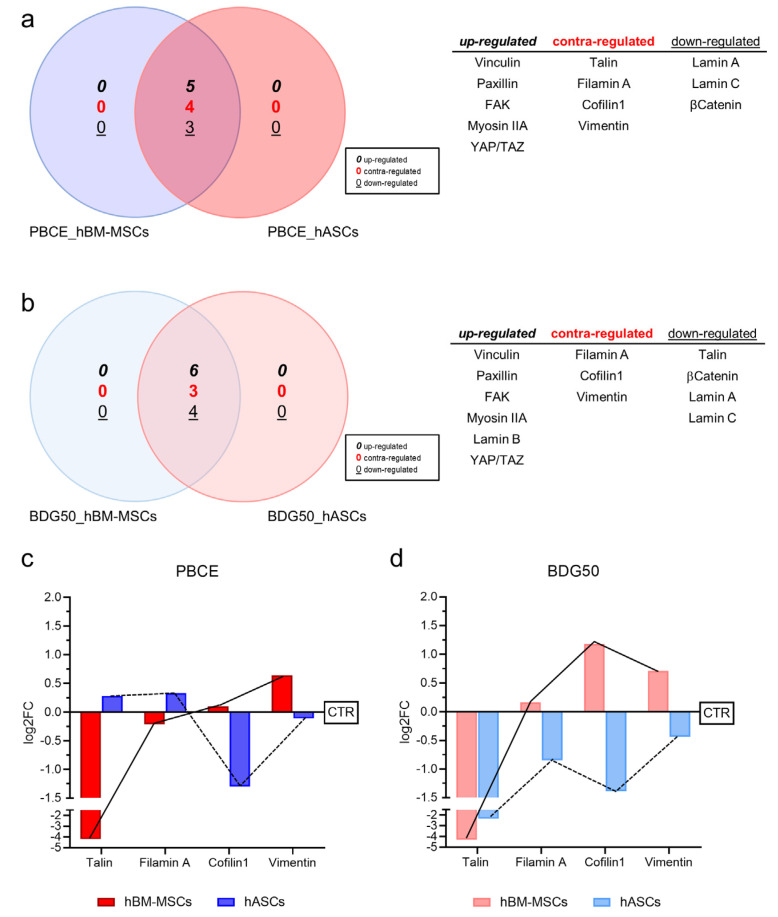
Two-set Venn diagram analysis of (**a**) hBM-MSCs and hASCs on PBCE homopolymer film and (**b**) hBM-MSCs and hASCs on BDG50 copolymer film. All proteins that are up− (italic font), down− (underlined font), and contra−regulated (red font) are indicated in a box shown next to each diagram. (**c**,**d**) Profile expression of (**c**) contra−regulated proteins in PBCE vs. CTR and (**d**) down−regulated Talin and contra−regulated proteins in BDG50 vs. CTR.

To shed light on the distinct responses of hBM-MSCs and hASCs to the PBCE and BDG50 films, we assessed the expression of proteins involved in the mechanotransduction process (Figure 1 and Figure 7–9). As previously mentioned, mechanotransduction involves a group of mechanotransducer proteins that can convert physical stimuli into biochemical signals. In this study, we focused on the expression of key proteins in the canonical mechanotransduction pathway, namely the focal adhesion (FA) complex, (Vinculin, Paxillin, Talin, FAK, and β-Catenin [2,58,59,60]); the Actin-linking proteins (Filamin A, Myosin IIA, and Cofilin1) necessary for the dynamicity of the actin microfilaments (F-Actin) [1,2,61]; and the nuclear lamins (Lamin A, Lamin B, Lamin C), which transmit the cytoskeleton signaling to the DNA [62,63,64].

Finally, we investigated the expression of the transcription factors YAP and TAZ, known for their roles as key mechanotransducers capable of transferring cytoskeletal stress to the nuclei and regulating cell function [65,66,67].

All data reported in Figure 7, Figure 8 and Figure 9 refer to the expression of the abovementioned proteins at D7, the time point at which the difference in both human adult mesenchymal/stromal multipotent cell shapes was clearly shown, and the cell number was sufficient for the experiments (Figure 7a,b). All histograms in the figures reflect densitometric analyses (mean of three different experiments) of related Western blotting experiments (for representative bands and densitometric analysis of the expression of every individual protein obtained by Western blotting, see Supplementary Figures S1–S4 and S6–S8).

Focal adhesion complex. The expression of each FA protein (Figures S1 and S2) gave rise to a specific FA complex composition profile in the hBM-MSC and hASC systems (Figure 7c,d).

Compared to control cells, in hBM-MSCs cultured on PBCE and BDG50, we found a significant increase in the expression of Vinculin (2.7- and 2.5-fold increase, respectively) and Paxillin (12- and 16-fold, respectively), whereas the expression of Talin was only slightly detectable (Figure S1). The expression of FAK, a protein involved in the assembly and maturation of the FA complex [68], was increased in cells on PBCE (66% > CTR) and to a greater extent on BDG50 (79% > CTR) (Figure S1). The expression of β-Catenin, a protein whose interaction with Vinculin responds to mechanical tension [69], was reduced in PBCE (46% < CTR) and BDG50 (85% < CTR) cultures (Figure S3a).

Similarly, in hASCs cultured on PBCE and BDG50, Vinculin and Paxillin expression was increased compared to control cells (Vinculin: 2.3 and 1.4-fold increase, PBCE; Paxillin: 3.2-fold and 2.4-fold increase, BDG50), while Talin expression was slightly increased on PBCE (8%) and reduced on BDG50 (82%) (Figure S2). FAK expression significantly increased on PBCE (2.9-fold > CTR) and to a lesser extent on BDG50 (1.4-fold > CTR) (Figure S2). β-Catenin expression was reduced in both PBCE (<31%) and BDG50 (<49%) cultures compared to control cells (Figure S3b).

Collectively, the Vinculin, Paxillin, Talin, and FAK proteins generated an expression profile that, starting from the expression of the Vinculin protein, known as the organizer of the mature FA complex [24,70], led to plots that were similar between hBM-MSCs and hASCs cultured on PBCE and on BDG50 (Figure 7c,d) and distinct compared to the related CTR culture counterparts (Figure 7c,d).

Actin-linking protein. The polymer films also influenced the expression of the Actin-linking proteins in both cell types (Figure 7e,f and Figure S4a,b). In the hBM-MSC system, the Myosin IIA expression was higher in cells on PBCE (2.6-fold increase) and to a lesser extent on BDG50 (1.8-fold increase) than in the control group (CTR). In contrast, Filamin A levels were reduced in hBM-MSCs on PBCE (17% < CTR), while they slightly increased on BDG50 (11% > CTR); Cofilin1 increased 2.4-fold on BDG50 compared to CTR, and to a lesser extent on PBCE (24% > CTR) (Figure 7e and Figure S4a).

In hASCs, the expression of Myosin IIA was significantly higher in the PBCE and BDG50 cultures (3.4- and 3.3-fold increase, respectively) when compared to the CTR culture. The expression of Filamin A was slightly increased in PBCE (26% > CTR) and highly reduced in hASCs on BDG50 (45% < CTR) (Figure 7f and Figure S4b); the expression of Cofilin1 dropped in the PBCE (<60%) and BDG50 cultures (<62%) compared to the CTR (Figure 7d and Figure S4b).

These findings confirm the role of Filamin A and Cofilin1 as key proteins with variable expression across systems (Figure 7e,f and Figure S4a,b).

Nucleoskeleton. No significant differences were observed in the measures of the nuclear shape index (NSI), nuclear roundness, and nuclear spread area between hBM-MSCs on PBCE, BDG50, and CTR (Figure S5), although all indicators showed a wide range of values on BDG50 (Figure S5a). These results diverged in hASCs, where the above descriptors showed a similar trend on PBCE and BDG50 compared to CTR (Figure S5b).

We further explored the effects of films on the hBM-MSCs’ and hASCs’ nucleoskeletons by evaluating the expression of nuclear Lamins A, C, and B (Figure 7g,h and Figure S6a,b).

In hBM-MSCs, Lamin A expression decreased on the BDG50 (68%) and PBCE (24%) films compared to CTR, while Lamin C expression remained unchanged and Lamin B expression increased slightly (15%) on BDG50 films compared to their expression in PBCE and CTR cultures (Figure S6a). In hASCs, Lamin A and C expression was significantly lower on PBCE (64% and 41%, respectively, compared to the CTR) and BDG50 (49% and 42%, respectively, compared to the CTR) (Figure S6b), whereas Lamin B expression was higher on PBCE (3.4-fold) and BDG50 (3.7-fold) compared to the CTR (Figure S6b).

Plotting together the expression of lamins, we observed that (i) Lamins A, C, and B had a similar trend of expression in CTR cells (Figure 7g,h); (ii) the lamins’ expression on PBCE was similar in hBM-MSCs, while, in hASCs, Lamin B was higher than Lamins A and C; in hBM-MSCs cultured on BDG50, Lamins C and B were higher compared to Lamin A, with differing expression profiles compared to hASCs, where Lamin B was greatly increased compared to Lamins A and C (Figure 7g,h).

Together, the results shown in Figure 7 and Figures S1–S4 and S6 highlight the differences in the expression of the overall mechanotransducer proteins in hASCs cultured on PBCE and BDG50 films compared to their hBM-MSC counterparts, thereby confirming the morphological differences between hBM-MSCs and hASCs cultured on polymer films (Figure 5 and Figure 6).

Effect of PBCE and BDG50 on YAP and TAZ. The analysis of the expression of the transcription factors YAP and TAZ in the polymer film cultures revealed the impact of the mechanical properties on the cell behavior Figure 8 and Figure S7a,b). YAP expression showed a significant increase in all cell–film cultures compared to the CTR (approximately 20-fold on BDG50 and 12-fold on PBCE for hBM-MSCs, and approximately 9-fold on PBCE and BDG50 for hASCs) (Figure 8a,b and Figure S7a,b). On the other hand, TAZ expression varied in hBM-MSCs on both films and in hASCs only on PBCE compared to the CTR. Specifically, TAZ levels decreased in hBM-MSCs on PBCE and BDG50 by approximately 60% less than for the CTR, while they significantly increased in hASCs on PBCE by 82% compared to the CTR and BDG50 (Figure 8a,b and Figure S7a,b). Plotting together YAP and TAZ, we observed an increase in the YAP/TAZ ratio in hBM-MSCs and hASCs seeded on PBCE and BDG50 compared to the CTR (Figure 8c). Interestingly, on PBCE and BDG50, hBM-MSCs had a higher YAP/TAZ ratio than hASCs, whereas the trend was the opposite in their CTR counterparts (Figure 8c).

#### 3.2.2. hBM-MSCs and hASCs Respond to PBCE and BDG50 by Activating Distinct Mechanotransduction Pathways

We used VennPlex, a computational analysis tool, to create a Venn diagram that grouped the mechanotransducer proteins analyzed in hBM-MSCs and hASCs cultured on PBCE and BDG50. This enabled us to identify the most relevant putative proteins in the mechanotransduction pathway(s) in BDG50 and PBCE cultures (Figure 9).

When comparing PBCE_hBM-MSCs and PBCE_hASCs, we found that five proteins were commonly upregulated (Vinculin, Paxillin, FAK, Myosin IIA, YAP/TAZ), three were downregulated (Lamin A, Lamin C, β-Catenin), and four were contra-regulated (Talin, Filamin A, Cofilin1, and Vimentin (see expression in Figure S8)) between systems (Figure 9a). Similarly, when comparing BDG50_hBM-MSCs and BDG50_hASCs, we identified six commonly upregulated proteins (Vinculin, Paxillin, FAK, Myosin IIA, Lamin B, YAP/TAZ), four downregulated proteins (Talin, β-Catenin, Lamin A, Lamin C), and three contra-regulated proteins (Filamin A, Cofilin1, Vimentin) (Figure 9b). Among the contra-regulated proteins, Cofilin1 and Vimentin were downregulated in hASCs and upregulated in hBM-MSCs in both films (Figure 9c,d). Filamin A was downregulated in hBM-MSCs and upregulated in hASCs on PBCE, and vice versa on BDG50 (Figure 9c,d). Talin was downregulated in h-BM-MSCs and upregulated in hASCs on PBCE, whereas it was downregulated in BDG50_hBM-MSCs and BDG50_hASCs (Figure 9c,d).

When correlating all the data in Figure 9, we uncovered that (i) the upregulated proteins (Vinculin, Paxillin, FAK, Myosin IIA, and YAP/TAZ) and the downregulated Lamins A and C were common to all groups investigated, proving that the BCE main moiety elicits this mechanotransduction pathway; (ii) the proteins Filamin A, Cofilin1, and Vimentin were contra-regulated in all groups, indicating that these proteins respond differently to the polymer based on the cell type; (iii) the protein Talin was contra-regulated in PBCE_hBM-MSCs–PBCE_hASCs and downregulated in BDG50_hBM-MSCs–BDG50_hASCs, suggesting that this protein responded differently to the different polymer film characteristics.

## 4. Discussion

In this work, we demonstrated that the BDG50 copolymer-based film triggered the reorganization of the cytoskeleton architecture and the acquisition of a new morphology in hBM-MSCs, which was drastically elongated and thinned out, compared to cells on the PBCE homopolymer-based film, where mesenchymal/stromal cells maintained the canonical mesenchymal fibroblast-like shape. These findings diverged from those showing a similar effect of PBCE and BDG50 films on hASCs, where the cells were elongated similarly to hBM-MSCs on the BDG50 film. The different cellular behaviors appeared early at D3 and were validated by the measurements of the computational cyto-morphometric descriptors, which further highlighted two clusters consisting of BDG50_hBM-MSCs versus PBCE_hBM-MSCs and control_hBM-MSCs (one side), and PBCE_hASCs–BDG50_hASCs versus control_hASCs (on the other side). We demonstrated that these results were the consequences of the specific mechanotransduction pathway(s) elicited by the properties of the PBCE and BDG50 films in both multipotent cell types.

The overall results validated the established notion that biomaterial properties can influence human adult mesenchymal/stromal multipotent cells’ fate (proliferation, morphology, cytoskeleton architecture, adhesion, differentiation [2,4,17,18,24,25,27,28,29,30,31,48,71,72]), but they primarily shed light on the mechanotransduction biochemical pathways that orchestrate the cellular behavior on both films.

Over the last few decades, it has been established that biomaterials possess some characteristics (e.g., surface micro/nanotopography, roughness/smoothness, hydrophilicity/hydrophobicity, stiffness, electricity) that enable them to mimic the cell microenvironment, which is a complex, geometric, organized structure consisting of fibrous proteins, polysaccharides, and soluble molecules, named the extracellular matrix (ECM) [4,11,24,30,72,73,74]. The ECM functions as a scaffold that provides mechanical support and drives biological signaling in cells and tissues, thus regulating cell homeostasis and phenotypes [75]. In this context, various material properties, such as stiffness, topology, surface chemistry, and particularly mechanical properties, are being explored to control the multiple signaling cascades involved in stem cell fate [18,24,25,30,33].

In this work, BDG50 films had higher hydrophilicity and reduced stiffness compared to PBCE, due to the incorporation of BDG co-units within the PBCE main chain.

Several authors correlated positively the increase in the hydrophilicity of the biomaterial surface and the increase in the cell proliferation rate and cellular adhesion [72,76,77], whereas variations in stiffness were associated with changes in cell morphology and cell spreading [78,79].

In our system, the difference in hydrophilicity between PBCE and BDG50 had no effect on cell proliferation, as demonstrated by the growth curves of the hBM-MSCs and hASCs on the films and their CTR counterparts. No alterations were observed also in the adhesion of hBM-MSCs and hASCs on both polymer films compared to the related CTRs. Supporting these findings are the comparable FA expression profiles between PBCE_hBM-MSCs and BDG50_hBM-MSCs, and PBCE_hASCs and BDG50_hASCs, as well as the expression of FAK, a protein sensitive to hydrophilicity, and Paxillin, a protein also sensitive to hydrophilicity and a putative substrate for FAK [80,81], which were always upregulated in all systems. Thus, the differences in the hydrophilicity of the two polymeric films may not have been directly involved in the observed cell shape changes. 

The impact of the different stiffness values of the PBCE and BDG50 films on hBM-MSCs and hASCs was more pronounced. 

It is well known that physiological tissues have different stiffness levels, with elastic moduli ranging from 0.5 to 1.5 kPa for soft tissue [44], such as fat or bone marrow, and increased values for hard tissue, such as bone. The latter is the stiffest tissue, where the elastic modulus ranges from 15 to 151 MPa, 17–20 GPa (longitudinal axis cortical bone) [44,45] and 6 to 13 GPa (transverse axis) [46].

In our system, the inclusion of the BDG unit into the PBCE film drastically decreased the elastic modulus from 560 MPa (PBCE) to 94 MPa (BDG50). In this regard, both films recapitulated the stiffness of hard tissue and therefore represent a good model to investigate the correlation between the expression of cell mechanotransduction and a high stiffness grade.

We observed a different effect of the film stiffness on the morphology and spreading area of the mesenchymal/stromal multipotent cells. hBM-MSCs only on the BDG50 film showed a changed morphology, reduced spread area, and increased spread index and roundness. The latter is considered the most sensitive cyto-morphometric descriptor of the energy interchange associated with morphological changes because of the cell–substrate interactions [79]. The above indicators and cell morphology were comparable in hBM-MSCs on PBCE and TCP or GC (elastic modulus ranging from 10^6^ to 10^7^ MPa and 69.74 ± 1.49 GPa, respectively) [82,83], indicating that these cells might be insensitive to these high stiffness levels.

Conversely, the hASCs showed a changed shape, reduced spread area, and increased spread index and roundness on both PBCE and BDG50 films, suggesting that these cells were similarly sensitive to both stiffness grades, 560MPa (PBCE) and 94MPa (BDG50), compared to the CTR.

The different responses of hBM-MSCs and hASCs might be dependent on the ontogenetic origin of both multipotent cell types, since hBM-MSCs reside in the bone marrow whereas hASCs reside in soft tissue; therefore, the former are more sensitive to the stiffness variations in both films compared to the latter [34,35,36,37,38,40,84].

Of note, our results on the different expression of the mechanotransduction proteins between cell–film systems might help to explain the different responses of hBM-MSCs and hASCs to PBCE and BDG50.

We followed the cell mechanotransduction pathway, beginning with (i) Vinculin, Talin, Paxillin, and FAK (focal adhesion complex), which are essential for cell interactions with the polymer film surface [1,2], and β-Catenin, which reacts to mechanical tension and interacts with Vinculin [69]; (ii) the dynamics of the cytoskeleton architecture (microtubules, F-Actin, and Vimentin) and the Actin-linking proteins (Cofilin1, FilaminA, and Myosin IIA), which gather the signals generated at the cell–film interface by the FA complex; and (iii) the nuclear lamins that transmit signals to the nucleus [1,2,85,86,87,88,89,90,91]. The latter, which acts as a mechanosensing machine, opens the nuclear pore complexes, allowing protein shuttling, including that of the mechanotransducer transcription factors YAP and TAZ [92].

The overall expression of the mechanotransducer proteins in PBCE_hBM-MSCs vs. PBCE_hASCs, and BDG50_hBM-MSCs vs. BDG50_hASCs, generated a map, in which the Vinculin, Paxillin, FAK, Myosin IIA, and YAP/TAZ proteins were always upregulated, while Lamin A, Lamin C, and β-Catenin were always downregulated, whereas the Filamin A, Cofilin1, and Vimentin proteins were contra-regulated between the two systems and Talin was downregulated in BDG50_hBM-MSCs and BDG50_hASCs, and contra-regulated in PBCE_hBM-MSCs and PBCE_hASCs.

The common upregulation and downregulation of the abovementioned proteins indicated that they were responsive to the BCE moieties present in both the homopolymer and copolymer films and might not have been directly involved in the different responses of cells on PBCE and BDG50, although their modulated expression was necessary for the transmission of the signals of the films to the cells.

The contra-regulated proteins are responsive to mechanical cues and interact directly or indirectly with F-Actin. Filamin A is a homodimer protein that guides the remodeling of F-Actin microfilaments, thus playing a role in determining cell shape and movement [93,94]. The protein is sensitive to mechanical cues, including stiffness variations, and also acts as a scaffold for signal transduction (e.g., binding with tyrosine kinase, phosphatases, GTPase) and FA proteins. Cofilin1 plays a central role in promoting the severing of F-Actin microfilaments, causing the depolymerization of F-Actin into G-Actin, and therefore is a key regulator of its dynamics [95]. Vimentin, a type III intermediate filament, is highly expressed in mesenchymal cells and has an essential role in maintaining cell integrity and stability even under mechanical stress, due to its mechanical properties (e.g., flexibility/stiffness) [96]. Moreover, it cooperates with F-Actin fibers in the maintenance of the cell shape. The focal adhesion protein Talin may act as a scaffold for the cells for the measurement of the extracellular rigidity, through binding with Vinculin and the F-Actin stress fibers [97,98]. Some authors have demonstrated that the absence of Talin impacts the capability of cells to determine whether they are on a soft or rigid substrate [97,98].

Collectively, our results demonstrated that the similar shape observed in hBM-MSCs on BDG50 and in hASCs on PBCE and BDG50 polymer films may be mainly attributed to the effects of the differences in film stiffness on the expression of Cofilin1, Vimentin, Filamin A, and Talin. Thus, we uncovered a tight, sensitive mechanism that allows hBM-MSCs to distinguish between PBCE and BDG50 compared to hASCs.

In conclusion, our study provides an innovative in vitro model to study the interactions of multipotential cells with highly stiff polymer films, which might mimic the stiffness of physiological hard tissue and therefore might be helpful in exploring the development of tissue engineering approaches for tissues such as bone. Mainly, our study emphasizes the cell-specific mechanotransduction response on films with different stiffness levels, thus providing new insights for the development of more effective biomaterials for biomedical applications or for innovative basic research on human adult mesenchymal/stromal multipotent cell biology.

## Figures and Tables

**Table 1:** List of primary antibodies.

Antibody	Dilution	Source	Company
Talin	1:1000	RABBIT	Cell Signaling Technology, Danvers, MA, USA
Paxillin	1:1000	RABBIT	Cell Signaling Technology, Danvers, MA, USA
Vinculin	1:1000	RABBIT	Abcam, Cambridge, UK
FAK	1:200	RABBIT	Santa Cruz Biotechnology, CA, USA
Β-Catenin	1:200	RABBIT	Elabscience, Houston, TX, USA
Vimentin	1:1000	RABBIT	Cell Signaling Technology, Danvers, MA, USA
Myosin IIA	1:1000	RABBIT	Santa Cruz Biotechnology, CA, USA
Filamin A	1:500	MOUSE	Santa Cruz Biotechnology, CA, USA
Cofilin1	1:200	RABBIT	Santa Cruz Biotechnology, CA, USA
Lamin A/C	1:1000	MOUSE	Cell Signaling Technology, Danvers, MA, USA
Lamin B	1:200	GOAT	Santa Cruz Biotechnology, CA, USA
YAP	1:1000	MOUSE	Cell Signaling Technology, Danvers, MA, USA
TAZ	1:1000	RABBIT	Cell Signaling Technology, Danvers, MA, USA
Actin	1:200	RABBIT	Sigma Aldrich, St. Louis, MI, USA

## Data Availability

Not applicable.

## Supplementary Materials

The following supporting information can be downloaded at: https://www.mdpi.com/article/10.3390/cells12131746/s1. Figure S1: Expression of FAs proteins in hBM-MSCs; Figure S2: Expression of FAs proteins in hASCs; Figure S3: βCatenin expression in hBM-MSCs and hASCs; Figure S4: Representative bands of Actin-linking proteins; Figure S5: Nuclear cyto-morphometric computational analysis of hBM-MSCs and hASCs; Figure S6: Expression of Lamin A, Lamin C, and Lamin B in hBM-MSCs and hASCs; Figure S7: Representative bands of mechanotransducer transcriptional factors; Figure S8: Vimentin expression in hBM-MSCs and hASCs.

## References

[1] Martino F., Perestrelo A.R., Vinarský V., Pagliari S., Forte G. (2018). Cellular mechanotransduction: From tension to function. Front. Physiol..

[2] Argentati C., Morena F., Tortorella I., Bazzucchi M., Porcellati S., Emiliani C., Martino S. (2019). Insight into Mechanobiology: How Stem Cells Feel Mechanical Forces and Orchestrate Biological Functions. Int. J. Mol. Sci..

[3] Raman N., Imran S.A.M., Noordin K.B.A.A., Zaman W.S.W.K., Nordin F. (2022). Mechanotransduction in Mesenchymal Stem Cells (MSCs) Differentiation: A Review. Int. J. Mol. Sci..

[4] Vining K.H., Mooney D.J. (2017). Mechanical forces direct stem cell behaviour in development and regeneration. Nat. Rev. Mol. Cell Biol..

[5] Ayad N.M.E., Kaushik S., Weaver V.M. (2019). Tissue mechanics, an important regulator of development and disease. Philos. Trans. R. Soc. B.

[6] Tortorella I., Argentati C., Emiliani C., Morena F., Martino S. (2022). Biochemical Pathways of Cellular Mechanosensing/Mechanotransduction and Their Role in Neurodegenerative Diseases Pathogenesis. Cells.

[7] Petzold J., Gentleman E. (2021). Intrinsic Mechanical Cues and Their Impact on Stem Cells and Embryogenesis. Front. Cell Dev. Biol..

[8] Alonso J.L., Goldmann W.H., Alonso J.L., Goldmann W.H. (2016). Cellular mechanotransduction. AIMS Biophys..

[9] Hayward M.K., Muncie J.M., Weaver V.M. (2021). Tissue mechanics in stem cell fate, development, and cancer. Dev. Cell.

[10] Hu D., Dong Z., Li B., Lu F., Li Y. (2022). Mechanical Force Directs Proliferation and Differentiation of Stem Cells. Tissue Eng. Part B Rev..

[11] Sun Y., Wan B., Wang R., Zhang B., Luo P., Wang D., Nie J.J., Chen D., Wu X. (2022). Mechanical Stimulation on Mesenchymal Stem Cells and Surrounding Microenvironments in Bone Regeneration: Regulations and Applications. Front. Cell Dev. Biol..

[12] Zhang X., Zhang S., Wang T. (2022). How the mechanical microenvironment of stem cell growth affects their differentiation: A review. Stem Cell Res. Ther..

[13] Dunn S.L., Olmedo M.L., Dunn S.L., Olmedo M.L. (2016). Mechanotransduction: Relevance to Physical Therapist Practice—Understanding Our Ability to Affect Genetic Expression Through Mechanical Forces. Phys. Ther..

[14] Frittoli E., Palamidessi A., Iannelli F., Zanardi F., Villa S., Barzaghi L., Abdo H., Cancila V., Beznoussenko G.V., Della Chiara G. (2023). Tissue fluidification promotes a cGAS–STING cytosolic DNA response in invasive breast cancer. Nat. Mater..

[15] Tassinari R., Olivi E., Cavallini C., Taglioli V., Zannini C., Marcuzzi M., Fedchenko O., Ventura C. (2023). Mechanobiology: A landscape for reinterpreting stem cell heterogeneity and regenerative potential in diseased tissues. iScience.

[16] Zhang Y., Habibovic P., Zhang Y., Habibovic P. (2022). Delivering Mechanical Stimulation to Cells: State of the Art in Materials and Devices Design. Adv. Mater..

[17] Argentati C., Dominici F., Morena F., Rallini M., Tortorella I., Ferrandez-Montero A., Pellegrino R.M., Ferrari B., Emiliani C., Lieblich M. (2022). Thermal treatment of magnesium particles in polylactic acid polymer films elicits the expression of osteogenic differentiation markers and lipidome profile remodeling in human adipose stem cells. Int. J. Biol. Macromol..

[18] Abbott R.D., Kaplan D.L. (2016). Engineering Biomaterials for Enhanced Tissue Regeneration. Curr. Stem Cell Rep..

[19] Li Y., Xu Z., Wang J., Pei X., Chen J., Wan Q. (2023). Alginate-based biomaterial-mediated regulation of macrophages in bone tissue engineering. Int. J. Biol. Macromol..

[20] Morille M., Toupet K., Montero-Menei C.N., Jorgensen C., Noël D. (2016). PLGA-based microcarriers induce mesenchymal stem cell chondrogenesis and stimulate cartilage repair in osteoarthritis. Biomaterials.

[21] Novikova L.N., Kolar M.K., Kingham P.J., Ullrich A., Oberhoffner S., Renardy M., Doser M., Müller E., Wiberg M., Novikov L.N. (2018). Trimethylene carbonate-caprolactone conduit with poly-p-dioxanone microfilaments to promote regeneration after spinal cord injury. Acta Biomater..

[22] Allur Subramanian S., Oh S., Mariadoss A.V.A., Chae S., Dhandapani S., Parasuraman P.S., Song S.Y., Woo C., Dong X., Choi J.Y. (2022). Tunable mechanical properties of Mo3Se3-poly vinyl alcohol-based/silk fibroin-based nanowire ensure the regeneration mechanism in tenocytes derived from human bone marrow stem cells. Int. J. Biol. Macromol..

[23] D’Angelo F., Armentano I., Cacciotti I., Tiribuzi R., Quattrocelli M., Del Gaudio C., Fortunati E., Saino E., Caraffa A., Cerulli G.G. (2012). Tuning multi/pluri-potent stem cell fate by electrospun poly(L-lactic acid)-calcium-deficient hydroxyapatite nanocomposite mats. Biomacromolecules.

[24] Morena F., Armentano I., Montanucci P., Argentati C., Fortunati E., Montesano S., Bicchi I., Pescara T., Pennoni I., Mattioli S. (2017). Design of a nanocomposite substrate inducing adult stem cell assembly and progression toward an Epiblast-like or Primitive Endoderm-like phenotype via mechanotransduction. Biomaterials.

[25] Morena F., Argentati C., Soccio M., Bicchi I., Luzi F., Torre L., Munari A., Emiliani C., Gigli M., Lotti N. (2020). Unpatterned Bioactive Poly(Butylene 1,4-Cyclohexanedicarboxylate)-Based Film Fast Induced Neuronal-Like Differentiation of Human Bone Marrow-Mesenchymal Stem Cells. Int. J. Mol. Sci..

[26] Yin Z., Wang J., Cui W., Tong C., Yin Z., Tong C., Wang J., Cui W. (2023). Advanced Biomaterials for Promoting Endometrial Regeneration. Adv. Healthc. Mater..

[27] Fu R.H., Wang Y.C., Liu S.P., Huang C.M., Kang Y.H., Tsai C.H., Shyu W.C., Lin S.Z. (2011). Differentiation of stem cells: Strategies for modifying surface biomaterials. Cell Transplant..

[28] Argentati C., Morena F., Fontana C., Tortorella I., Emiliani C., Latterini L., Zampini G., Martino S. (2021). Functionalized Silica Star-Shaped Nanoparticles and Human Mesenchymal Stem Cells: An In Vitro Model. Nanomaterials.

[29] Ben Abdeljawad M., Carette X., Argentati C., Martino S., Gonon M.F., Odent J., Morena F., Mincheva R., Raquez J.M. (2021). Interfacial Compatibilization into PLA/Mg Composites for Improved In Vitro Bioactivity and Stem Cell Adhesion. Molecules.

[30] Argentati C., Morena F., Montanucci P., Rallini M., Basta G., Calabrese N., Calafiore R., Cordellini M., Emiliani C., Armentano I. (2018). Surface Hydrophilicity of Poly(l-Lactide) Acid Polymer Film Changes the Human Adult Adipose Stem Cell Architecture. Polymer.

[31] Naqvi S.M., McNamara L.M. (2020). Stem Cell Mechanobiology and the Role of Biomaterials in Governing Mechanotransduction and Matrix Production for Tissue Regeneration. Front. Bioeng. Biotechnol..

[32] Abdulghani S., Mitchell G.R. (2019). Biomaterials for In Situ Tissue Regeneration: A Review. Biomolecules.

[33] Williams D.F. (2019). Challenges with the Development of Biomaterials for Sustainable Tissue Engineering. Front. Bioeng. Biotechnol..

[34] Dominici M., Le Blanc K., Mueller I., Slaper-Cortenbach I., Marini F.C., Krause D.S., Deans R.J., Keating A., Prockop D.J., Horwitz E.M. (2006). Minimal criteria for defining multipotent mesenchymal stromal cells. The International Society for Cellular Therapy position statement. Cytotherapy.

[35] Viswanathan S., Shi Y., Galipeau J., Krampera M., Leblanc K., Martin I., Nolta J., Phinney D.G., Sensebe L. (2019). Mesenchymal stem versus stromal cells: International Society for Cell & Gene Therapy (ISCT^®^) Mesenchymal Stromal Cell committee position statement on nomenclature. Cytotherapy.

[36] Merrick D., Sakers A., Irgebay Z., Okada C., Calvert C., Morley M.P., Percec I., Seale P. (2019). Identification of a mesenchymal progenitor cell hierarchy in adipose tissue. Science.

[37] Argentati C., Tortorella I., Bazzucchi M., Morena F., Martino S. (2020). Harnessing the Potential of Stem Cells for Disease Modeling: Progress and Promises. J. Pers. Med..

[38] Hmadcha A., Martin-Montalvo A., Gauthier B.R., Soria B., Capilla-Gonzalez V. (2020). Therapeutic Potential of Mesenchymal Stem Cells for Cancer Therapy. Front. Bioeng. Biotechnol..

[39] Chan C.K.F., Seo E.Y., Chen J.Y., Lo D., McArdle A., Sinha R., Tevlin R., Seita J., Vincent-Tompkins J., Wearda T. (2015). Identification and specification of the mouse skeletal stem cell. Cell.

[40] Argentati C., Morena F., Bazzucchi M., Armentano I., Emiliani C., Martino S. (2018). Adipose Stem Cell Translational Applications: From Bench-to-Bedside. Int. J. Mol. Sci..

[41] Gigli M., Lotti N., Vercellino M., Visai L., Munari A. (2014). Novel ether-linkages containing aliphatic copolyesters of poly(butylene 1,4-cyclohexanedicarboxylate) as promising candidates for biomedical applications. Mater. Sci. Eng. C. Mater. Biol. Appl..

[42] Bloise N., Berardi E., Gualandi C., Zaghi E., Gigli M., Duelen R., Ceccarelli G., Cortesi E.E., Costamagna D., Bruni G. (2018). Ether-Oxygen Containing Electrospun Microfibrous and Sub-Microfibrous Scaffolds Based on Poly(butylene 1,4-cyclohexanedicarboxylate) for Skeletal Muscle Tissue Engineering. Int. J. Mol. Sci..

[43] Fusaro L., Gualandi C., Antonioli D., Soccio M., Liguori A., Laus M., Lotti N., Boccafoschi F., Focarete M.L. (2020). Elastomeric Electrospun Scaffolds of a Biodegradable Aliphatic Copolyester Containing PEG-Like Sequences for Dynamic Culture of Human Endothelial Cells. Biomolecules.

[44] Handorf A.M., Zhou Y., Halanski M.A., Li W.J. (2015). Tissue Stiffness Dictates Development, Homeostasis, and Disease Progression. Organogenesis.

[45] Chen X., Hughes R., Mullin N., Hawkins R.J., Holen I., Brown N.J., Hobbs J.K. (2020). Mechanical Heterogeneity in the Bone Microenvironment as Characterized by Atomic Force Microscopy. Biophys. J..

[46] Porter J.R., Ruckh T.T., Popat K.C. (2009). Bone tissue engineering: A review in bone biomimetics and drug delivery strategies. Biotechnol. Prog..

[47] Morena F., Argentati C., Calzoni E., Cordellini M., Emiliani C., D’Angelo F., Martino S. (2016). Ex-Vivo Tissues Engineering Modeling for Reconstructive Surgery Using Human Adult Adipose Stem Cells and Polymeric Nanostructured Matrix. Nanomaterials.

[48] Luzi F., Tortorella I., Di Michele A., Dominici F., Argentati C., Morena F., Torre L., Puglia D., Martino S. (2020). Novel Nanocomposite PLA Films with Lignin/Zinc Oxide Hybrids: Design, Characterization, Interaction with Mesenchymal Stem Cells. Nanomaterials.

[49] Bicchi I., Morena F., Argentati C., Nodari L.R., Emiliani C., Gelati M., Vescovi A.L., Martino S. (2021). Storage of mutant human sod1 in non-neural cells from the type-1 amyotrophic lateral sclerosis ratg93a model correlated with the lysosomes’ dysfunction. Biomedicines.

[50] Schindelin J., Arganda-Carreras I., Frise E., Kaynig V., Longair M., Pietzsch T., Preibisch S., Rueden C., Saalfeld S., Schmid B. (2012). Fiji: An open-source platform for biological-image analysis. Nat. Methods.

[51] Schwendy M., Unger R.E., Bonn M., Parekh S.H. (2019). Automated cell segmentation in FIJI^®^ using the DRAQ5 nuclear dye. BMC Bioinform..

[52] Martino S., Emiliani C., Tancini B., Severini G.M., Chigorno V., Bordignon C., Sonnino S., Orlacchio A. (2002). Absence of metabolic cross-correction in Tay-Sachs cells: Implications for gene therapy. J. Biol. Chem..

[53] Morena F., Argentati C., Trotta R., Crispoltoni L., Stabile A., Pistilli A., di Baldassarre A., Calafiore R., Montanucci P., Basta G. (2017). A Comparison of Lysosomal Enzymes Expression Levels in Peripheral Blood of Mild- and Severe-Alzheimer’s Disease and MCI Patients: Implications for Regenerative Medicine Approaches. Int. J. Mol. Sci..

[54] Cai H., Chen H., Yi T., Daimon C.M., Boyle J.P., Peers C., Maudsley S., Martin B. (2013). VennPlex–A Novel Venn Diagram Program for Comparing and Visualizing Datasets with Differentially Regulated Datapoints. PLoS ONE.

[55] Atherton P., Lausecker F., Carisey A., Gilmore A., Critchley D., Barsukov I., Ballestrem C. (2019). Force-independent interactions of talin and vinculin govern integrin-mediated mechanotransduction. bioRxiv.

[56] Sanghvi-Shah R., Weber G.F. (2017). Intermediate filaments at the junction of mechanotransduction, migration, and development. Front. Cell Dev. Biol..

[57] Kidd M.E., Shumaker D.K., Ridge K.M. (2014). The role of vimentin intermediate filaments in the progression of lung cancer. Am. J. Respir. Cell Mol. Biol..

[58] Legerstee K., Geverts B., Slotman J.A., Houtsmuller A.B. (2019). Dynamics and distribution of paxillin, vinculin, zyxin and VASP depend on focal adhesion location and orientation. Sci. Rep..

[59] Grashoff C., Hoffman B.D., Brenner M.D., Zhou R., Parsons M., Yang M.T., McLean M.A., Sligar S.G., Chen C.S., Ha T. (2010). Measuring mechanical tension across vinculin reveals regulation of focal adhesion dynamics. Nature.

[60] Stutchbury B., Atherton P., Tsang R., Wang D.Y., Ballestrem C. (2017). Distinct focal adhesion protein modules control different aspects of mechanotransduction. J. Cell Sci..

[61] Stachowiak M.R., Smith M.A., Blankman E., Chapin L.M., Balcioglu H.E., Wang S., Beckerle M.C., O’Shaughnessy B. (2014). A mechanical-biochemical feedback loop regulates remodeling in the actin cytoskeleton. Proc. Natl. Acad. Sci. USA.

[62] Malashicheva A., Perepelina K. (2021). Diversity of Nuclear Lamin A/C Action as a Key to Tissue-Specific Regulation of Cellular Identity in Health and Disease. Front. Cell Dev. Biol..

[63] Pascual-Reguant L., Blanco E., Galan S., Le Dily F., Cuartero Y., Serra-Bardenys G., Di Carlo V., Iturbide A., Cebrià-Costa J.P., Nonell L. (2018). Lamin B1 mapping reveals the existence of dynamic and functional euchromatin lamin B1 domains. Nat. Commun..

[64] Bouzid T., Kim E., Riehl B.D., Esfahani A.M., Rosenbohm J., Yang R., Duan B., Lim J.Y. (2019). The LINC complex, mechanotransduction, and mesenchymal stem cell function and fate. J. Biol. Eng..

[65] Cai X., Wang K.C., Meng Z. (2021). Mechanoregulation of YAP and TAZ in Cellular Homeostasis and Disease Progression. Front. Cell Dev. Biol..

[66] Panciera T., Azzolin L., Cordenonsi M., Piccolo S. (2017). Mechanobiology of YAP and TAZ in physiology and disease. Nat. Rev. Mol. Cell Biol..

[67] Dupont S., Morsut L., Aragona M., Enzo E., Giulitti S., Cordenonsi M., Zanconato F., Le Digabel J., Forcato M., Bicciato S. (2011). Role of YAP/TAZ in mechanotransduction. Nature.

[68] Mitra S.K., Hanson D.A., Schlaepfer D.D. (2005). Focal adhesion kinase: In command and control of cell motility. Nat. Rev. Mol. Cell Biol..

[69] Peng X., Cuff L.E., Lawton C.D., DeMali K.A. (2010). Vinculin regulates cell-surface E-cadherin expression by binding to beta-catenin. J. Cell Sci..

[70] Carisey A., Tsang R., Greiner A.M., Nijenhuis N., Heath N., Nazgiewicz A., Kemkemer R., Derby B., Spatz J., Ballestrem C. (2013). Vinculin regulates the recruitment and release of core focal adhesion proteins in a force-dependent manner. Curr. Biol..

[71] Donnelly H., Salmeron-Sanchez M., Dalby M.J. (2018). Designing stem cell niches for differentiation and self-renewal. J. R. Soc. Interface.

[72] Kerch G. (2018). Polymer hydration and stiffness at biointerfaces and related cellular processes. Nanomedicine.

[73] Romani P., Valcarcel-Jimenez L., Frezza C., Dupont S. (2021). Crosstalk between mechanotransduction and metabolism. Nat. Rev. Mol. Cell Biol..

[74] Willerth S.M., Sakiyama-Elbert S.E. (2019). Combining Stem Cells and Biomaterial Scaffolds for Constructing Tissues and Cell Delivery. StemJournal.

[75] Fernandes T.G. (2022). Design and Fabrication of Artificial Stem Cell Niches. Bioengineering.

[76] Zan F., Wei Q., Fang L., Xian M., Ke Y., Wu G. (2020). Role of Stiffness versus Wettability in Regulating Cell Behaviors on Polymeric Surfaces. ACS Biomater. Sci. Eng..

[77] Fan H., Guo Z. (2020). Bioinspired surfaces with wettability: Biomolecule adhesion behaviors. Biomater. Sci..

[78] Janmey P.A., Fletcher D.A., Reinhart-King C.A. (2020). Stiffness Sensing by Cells. Physiol. Rev..

[79] Chiang M.Y.M., Yangben Y., Lin N.J., Zhong J.L., Yang L. (2013). Relationships among cell morphology, intrinsic cell stiffness and cell-substrate interactions. Biomaterials.

[80] Abedi H., Zachary I. (1997). Vascular endothelial growth factor stimulates tyrosine phosphorylation and recruitment to new focal adhesions of focal adhesion kinase and paxillin in endothelial cells. J. Biol. Chem..

[81] Xu J., Cui Y., Liu M., An Z., Li K., Gu X., Li P., Fan Y. (2023). Enhanced hydrophilicity of one-step electrosprayed red blood cell-like PLGA microparticles by block polymer PLGA-PEG-PLGA with excellent magnetic-luminescent bifunction and affinity to HUVECs. Eur. Polym. J..

[82] Fekete N., Béland A.V., Campbell K., Clark S.L., Hoesli C.A. (2018). Bags versus flasks: A comparison of cell culture systems for the production of dendritic cell–based immunotherapies. Transfusion.

[83] Wang P.-H., Mao B.-H., Mai Nguyen Thi K., Tang M.-J., Kamm R.D., Tu T.-Y. (2023). The interface stiffness and topographic feature dictate interfacial invasiveness of cancer spheroids. Biofabrication.

[84] Du J., Wang Z., Liu X., Hu C., Yarema K.J., Jia X. (2023). Improving Schwann Cell Differentiation from Human Adipose Stem Cells with Metabolic Glycoengineering. Cells.

[85] Gao J., Nakamura F. (2022). Actin-Associated Proteins and Small Molecules Targeting the Actin Cytoskeleton. Int. J. Mol. Sci..

[86] Pollard T.D. (2016). Actin and Actin-Binding Proteins. Cold Spring Harb. Perspect. Biol..

[87] Wiggan O., Shaw A.E., DeLuca J.G., Bamburg J.R. (2012). ADF/cofilin regulates actomyosin assembly through competitive inhibition of myosin II binding to F-actin. Dev. Cell.

[88] Bugyi B., Carlier M.F. (2010). Control of actin filament treadmilling in cell motility. Annu. Rev. Biophys..

[89] Ono S. (2020). Cofilin-induced structural changes in actin filaments stay local. Proc. Natl. Acad. Sci. USA.

[90] Tanaka K., Takeda S., Mitsuoka K., Oda T., Kimura-Sakiyama C., Maéda Y., Narita A. (2018). Structural basis for cofilin binding and actin filament disassembly. Nat. Commun..

[91] Hayakawa K., Tatsumi H., Sokabe M. (2011). Actin filaments function as a tension sensor by tension-dependent binding of cofilin to the filament. J. Cell Biol..

[92] Dickmanns A., Kehlenbach R.H., Fahrenkrog B. (2015). Nuclear Pore Complexes and Nucleocytoplasmic Transport: From Structure to Function to Disease. Int. Rev. Cell Mol. Biol..

[93] Byfield F.J., Wen Q., Levental I., Nordstrom K., Arratia P.E., Miller R.T., Janmey P.A. (2009). Absence of Filamin A Prevents Cells from Responding to Stiffness Gradients on Gels Coated with Collagen but not Fibronectin. Biophys. J..

[94] Zhou J., Kang X., An H., Lv Y., Liu X. (2021). The function and pathogenic mechanism of filamin A. Gene.

[95] Bamburg J.R., Bernstein B.W. (2010). Roles of ADF/cofilin in actin polymerization and beyond. F1000 Biol. Rep..

[96] Goldman R.D., Khuon S., Chou Y.H., Opal P., Steinert P.M. (1996). The function of intermediate filaments in cell shape and cytoskeletal integrity. J. Cell Biol..

[97] Kumar A., Ouyang M., Van den Dries K., McGhee E.J., Tanaka K., Anderson M.D., Groisman A., Goult B.T., Anderson K.I., Schwartz M.A. (2016). Talin tension sensor reveals novel features of focal adhesion force transmission and mechanosensitivity. J. Cell Biol..

[98] Austen K., Ringer P., Mehlich A., Chrostek-Grashoff A., Kluger C., Klingner C., Sabass B., Zent R., Rief M., Grashoff C. (2015). Extracellular rigidity sensing by talin isoform-specific mechanical linkages. Nat. Cell Biol..

