# Supplementary material for: Tight Regulation of Mechanotransducer Proteins Distinguishes the Response of Adult Multipotent Mesenchymal Cells on PBCE-Derivative Polymer Films with Different Hydrophilicity and Stiffness
Source: Cells. 2023 Jun 29;12(13):1746. doi: 10.3390/cells12131746 (PMC10341130; doi:10.3390/cells12131746)
Supplement: Supplementary file 1 [file cells-12-01746-s001.zip › cells-2439393-supplementary.pdf]

The supplementary file includes Supplementary Figures S1-S8 with related captions:

Figure S1: Expression of FAs proteins in hBM-MSCs;

Figure S2: Expression of FAs proteins in hASCs;

Figure S3: βCatenin expression in hBM-MSCs and hASCs;

Figure S4: Representative bands of Actin-linking proteins;

Figure S5: Nuclear cyto-morphometric computational analysis of hBM-MSCs and hASCs;

Figure S6: Expression of Lamin A, Lamin C, and Lamin B in hBM-MSCs and hASCs;

Figure S7: Representative bands of mechanotrasducer transcriptional factors;

Figure S8: Vimentin expression in hBM-MSCs and hASCs.

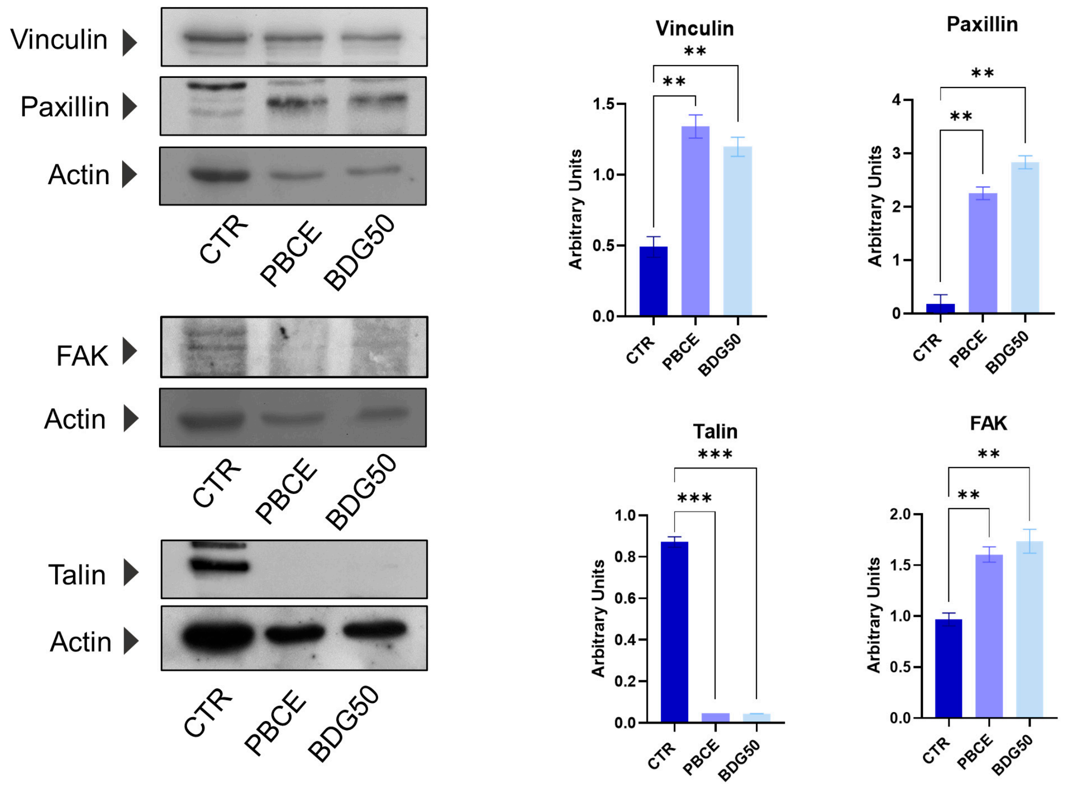

Figure S1. Expression of FAs proteins in hBM-MSCs on TCP (CTR), PBCE and BDG50 films at D7. Representative bands and relative densitometric analysis of 3 independent experiments of western blot showing protein expressions of Vinculin, Talin, Paxillin and FAK. Results were expressed as the mean ± SD of three independent experiments, each in triplicates. **$p < 0.01$, ***$p < 0.001$.

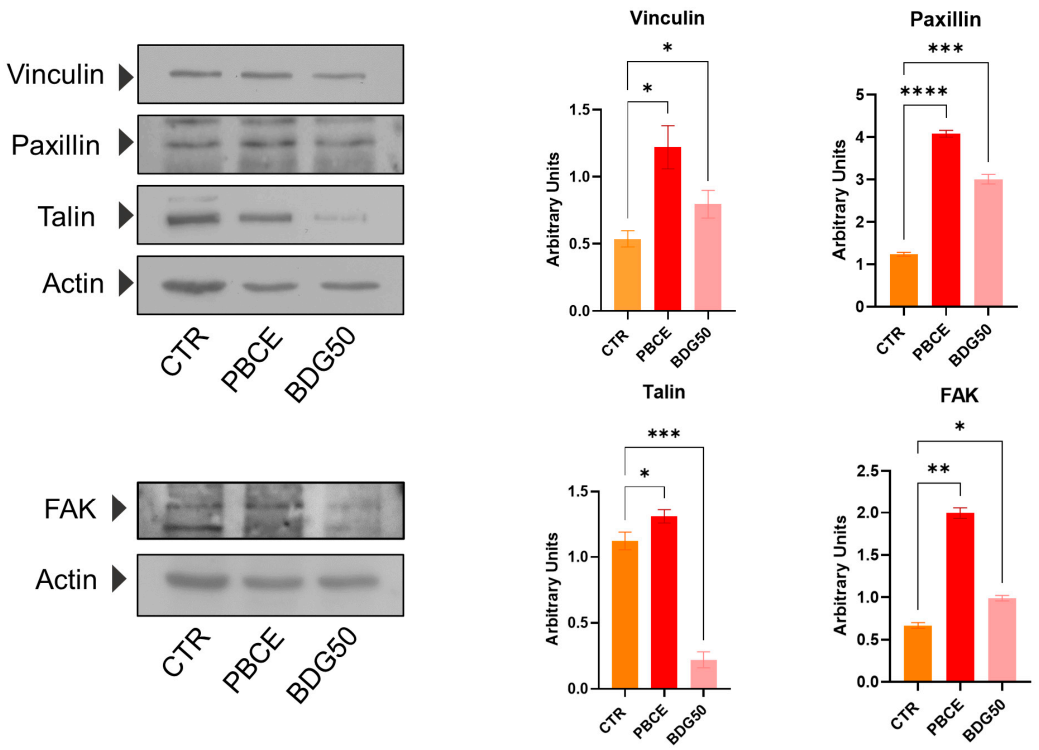

Figure S2. Expression of FAs proteins in hASCs on TCP (CTR), PBCE and BDG50 films at D7. Representative bands and relative densitometric analysis of 3 independent experiments of western blot showing protein expressions of Vinculin, Talin, Paxillin and FAK. Results were expressed as the mean ± SD of three independent experiments, each in triplicates. *$p < 0.05$, **$p < 0.01$, ***$p < 0.001$, ****$p < 0.0001$.

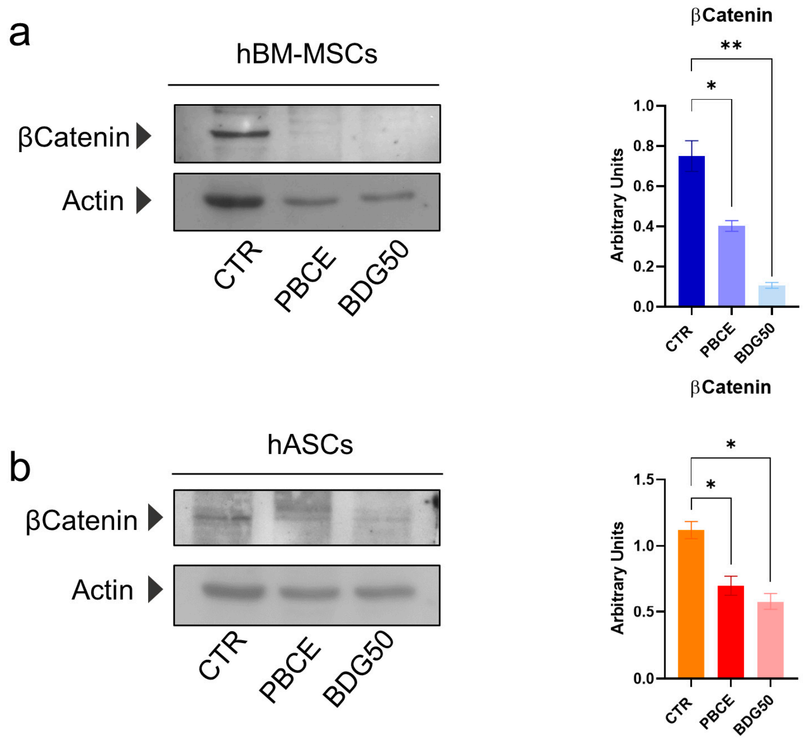

Figure S3. Expression of βCatenin in hBM-MSCs **a)** and hASCs **b)** on PBCE, BDG50 films and CTR at D7 and relative densitometric analysis. Results were expressed as the mean ± SD of three independent experiments, each in triplicates. *$p < 0.05$, **$p < 0.01$.

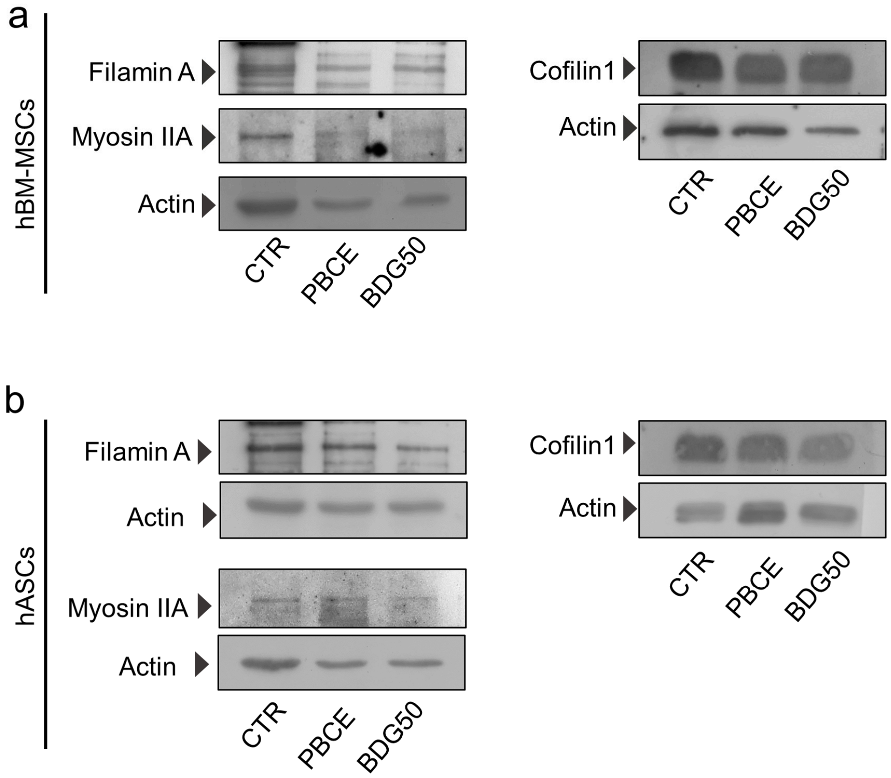

Figure S4. Representative bands of Actin-linking proteins in **a)** hBM-MSCs and **b)** hASCs on TCP (CTR), PBCE and BDG50 respectively at D7. Representative bands of 3 independent experiments of western blot showing protein expressions of Actin-linking proteins: Filamin A, Myosin IIA and Cofilin1.

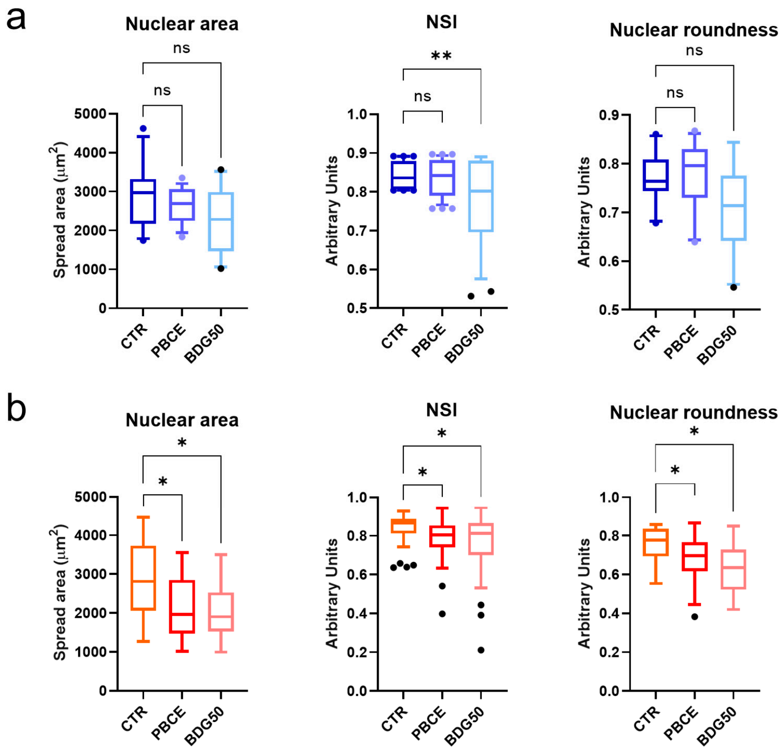

Figure S5. Nuclear area, Nuclear Shape Index (NSI) and Nuclear roundness of hBM-MSCs (a) and hASCs (b) on CTR, PBCE and BDG50 films. Data are represented as box and whiskers (min to max) with Kruskall–Wallis test and Dunn's multiple comparison test. * $p < 0.05$, ** $p < 0.01$.

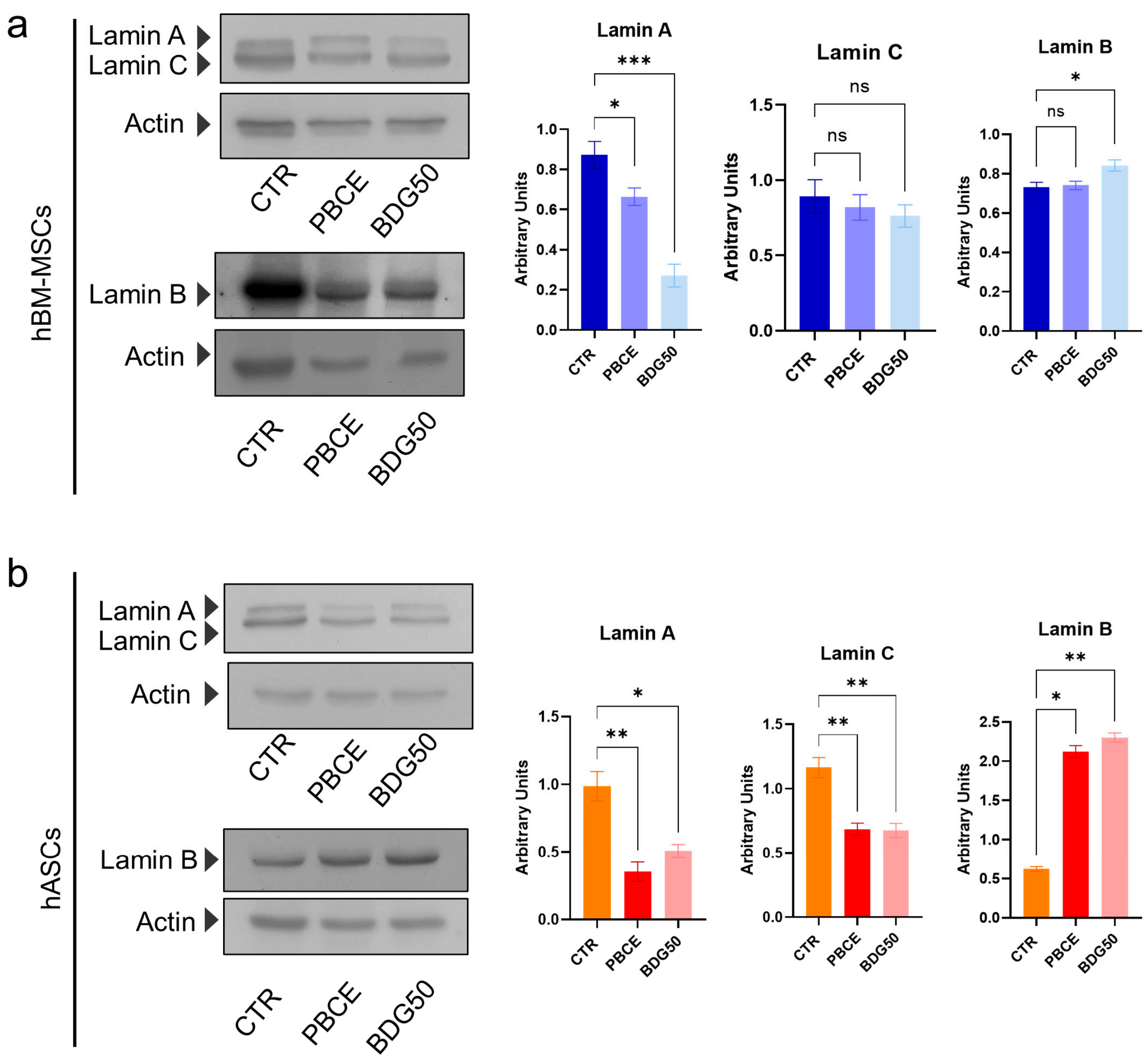

Figure S6. Expression of Lamin A, Lamin C and Lamin B in **a)** hBM-MSCs and **b)** hASCs on TCP (CTR), PBCE and BDG50 rispectively at D7. Representative bands and relative densitometric analysis of 3 independent experiments of western blot showing protein expressions of Nucleoskeleton proteins: Lamin A, Lamin C and Lamin B. Results were expressed as the mean ± SD of three independent experiments, each in triplicates. *p < 0.05, **p < 0.01, ***p < 0.001.

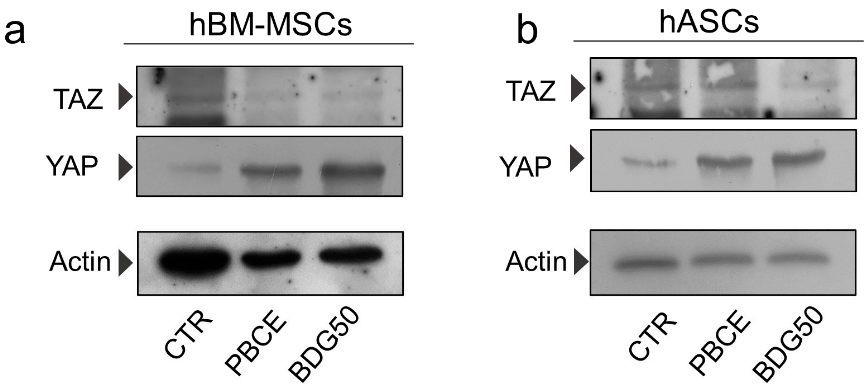

Figure S7. Representative bands of mechanotrasducer transcriptional factors. Yap and TAZ **a)** in hBM-MSCs and **b)** hASCs on TCP (CTR), PBCE and BDG50 respectively at D7.

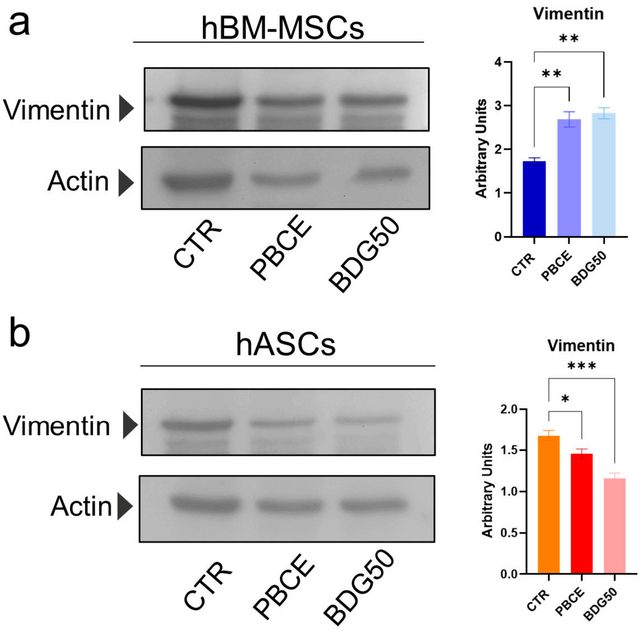

Figure S8. Vimentin expression in hBM-MSCs **a)**, and hASCs **b)** on PBCE, BDG50 films and CTR at D7 and relative densitometric analysis. Results were expressed as mean ± SD of three independent experiments, each in triplicates. *p<0.05, **p<0.01., ***p < 0.001.

The Vimentin expression was increased in hBM-MSCs on both PBCE (55% higher than control) and BDG50 (63% higher than control) films, whereas was reduced in hASCs on both PBCE (13% less than the control) and BDG50 (30% less than the control) films.

These results support the involvement of IFs in the morphological changes in both multipotent cells.
